# One-Dimensional Nanostructured Oxide Chemoresistive Sensors

**DOI:** 10.1021/acs.langmuir.0c00701

**Published:** 2020-05-26

**Authors:** Navpreet Kaur, Mandeep Singh, Elisabetta Comini

**Affiliations:** Sensor Laboratory, University of Brescia, Via D. Valotti 9, 25133 Brescia, Italy

## Abstract

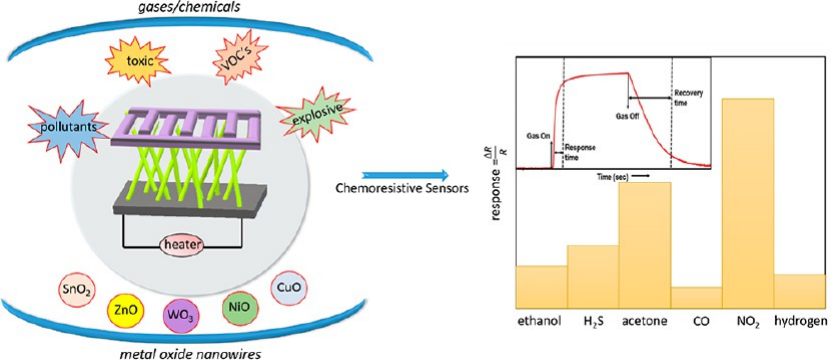

Day by day, the demand for portable, low cost, and efficient chemical/gas-sensing devices is increasing due to worldwide industrial growth for various purposes such as environmental monitoring and health care. To fulfill this demand, nanostructured metal oxides can be used as active materials for chemical/gas sensors due to their high crystallinity, remarkable physical/chemical properties, ease of synthesis, and low cost. In particular, (1D) one-dimensional metal oxides nanostructures, such as nanowires, exhibit a fast response, selectivity, and stability due to their high surface-to-volume ratio, well-defined crystal orientations, controlled unidirectional electrical properties, and self-heating phenomenon. Moreover, with the availability of large-scale production methods for nanowire growth such as thermal oxidation and evaporation–condensation growth, the development of highly efficient, low cost, portable, and stable chemical sensing devices is possible. In the last two decades, tremendous advances have been achieved in 1D nanostructured gas sensors ever since the pioneering work by Comini on the development of a SnO_2_ nanobelt for gas sensor applications in 2002, which is one such example from which many researchers began to explore the field of 1D-nanostructure-based chemical/gas sensors. The Sensor Laboratory (University of Brescia) has made major contributions to the field of metal oxide nanowire chemical/gas-sensing devices. Over the years, different metal oxides such as SnO_2_, ZnO, WO_3_, NiO, CuO, and their heterostructures have been grown for their nanowire morphology and successfully integrated into chemoresistive gas-sensing devices. Hence in this invited feature article, Sensor Laboratory research on the synthesis of metal oxide nanowires and novel heterostructures and their characterization and gas-sensing performance during exposure to different gas analytes has been presented. Moreover, some new strategies such as branched-like nanowire heterostructures and core–shell nanowire structures adopted to enhance the performance of nanowire-based chemical sensor are presented in detail.

## Introduction

From its beginning, the electronics industry was ruled by silicon. In parallel to its evolution, there is a continuous effort to find alternative materials due to the high cost of silicon-based electronic devices and most importantly to avoid relying on a single material. Thus, as an alternatives, many materials such as organic semiconductors^1^ and pervoskites^2^ have been explored by researchers over the years. In 2010, when the Nobel Prize in Physics was announced, graphene^3^ was immediately considered to be the material of the century. However, among all of these, metal oxides (MOXs) were silently doing their job, and nowadays they are used in many modern electronic device applications such as thin film transistors,^4^ chemical sensors,^5^ biosensors,^6^ and solar cells.^7^ The reason behind their success is high stability, abundance on earth, and remarkable chemical/physical properties that can be tuned according to the requirements for particular applications.

Among the successful exploitation of MOXs, there are commercially available indium gallium zinc oxide (IGZO)-based high-mobility transistors.^8,9^ MOXs are also considered to be potential candidates for future transparent electronic devices due to their high band gap in the ultraviolet region that makes them transparent in the visible region of the spectrum.^10−12^

Another important area of application in which MOXs are immensely explored is chemical/gas sensors due to their properties such as low cost, compact size, ease of fabrication, and high abundance on the earth’s crust.^13,14^ For example, tin dioxide (SnO_2_) is one of the most explored materials in the field of gas sensors.^15−17^ A chemical sensor is a device that convert a chemical signal obtained via the interaction of a chemical analyte with an active sensing materials (such as MOX) into a measurable signal (optical, electrical, and magnetic etc.).^18^ In the last two decades, chemoresistive sensors (measurable signal: resistance or conductance) have been extensively investigated to detect a large number of chemical analytes such as volatile organic compounds,^19^ toxic gases,^20^ explosives,^21^ and environment pollutants.^22^ The performance of these sensing devices largely depends upon the microstructural properties of the active sensing material. In this regard, nanostructured metal oxide-based gas sensors are attracting attention due to their fast response and high sensitivity.^23−25^ However, the selectivity of these devices is still a major issue, and many different strategies such as surface functionalization have been explored to limit or overcome this challenging issue.^26,27^ Moreover, MOXs in nanostructured form possess a high surface-to-volume ratio, a higher degree of crystallinity, and better stoichiometry.^23^ Another important aspect of MOXs is the possibility to grow them with different morphologies such as nanowires,^5^ nanobelts,^28^ and nanodots.^29^ These MOX nanostructures can be grown by using physical,^30^ chemical,^31^ and solution-processable techniques.^32^

Among these nanostructured forms, 1D MOX nanowires (NWs) possess well-defined crystal orientations and single crystallinity that leads to controlled reactions and increased stability of the sensing devices based on them.^23,33^ The MOX NW gas sensors had faster response dynamics because there was no need for gas diffusion prior to surface reactions. Moreover, very interesting effects such as self-heating, which can be exploited in gas sensing, are effective only for nanowire morphology.^5,34^ For recent developments in nanostructured metal oxide gas sensors, the reader can also refer to other interesting review articles.^35−37^

In the field of MOX NW-based gas sensors, the Sensor Laboratory (University of Brescia) has done pioneering work concerning the growth, integration, functional characterization, and improvements of the MOX sensing performance. We have grown MOX NWs such as ZnO, SnO_2_, WO_3_, TiO_2_, CuO, and NiO using different techniques and procedures. These techniques are mainly based on two principles: thermal oxidation and vapor-phase growth using vapor–solid (VS) and vapor–liquid–solid (VLS) mechanisms. In all of these cases, laboratory-made equipment has been developed by the Sensor Laboratory.

In this invited feature article, the contributions of the Sensor Laboratory to the development of MOX NW-based chemical/gas sensors will be presented. In particular, the working principles of chemoresistive sensors, the gas-sensing mechanism, the device structure, and the techniques used for nanowire synthesis and their growth mechanism will be described. Moreover, their functional properties, measured using different techniques, will be discussed. In the final sections, their sensing performance and new strategies to enhance their performance will be presented.

## Metal Oxide Gas-Sensing Mechanism and Device Structure of a Conductometric Gas Sensor and Their Working Principles

### Gas-Sensing Mechanism

**Figure 1:**
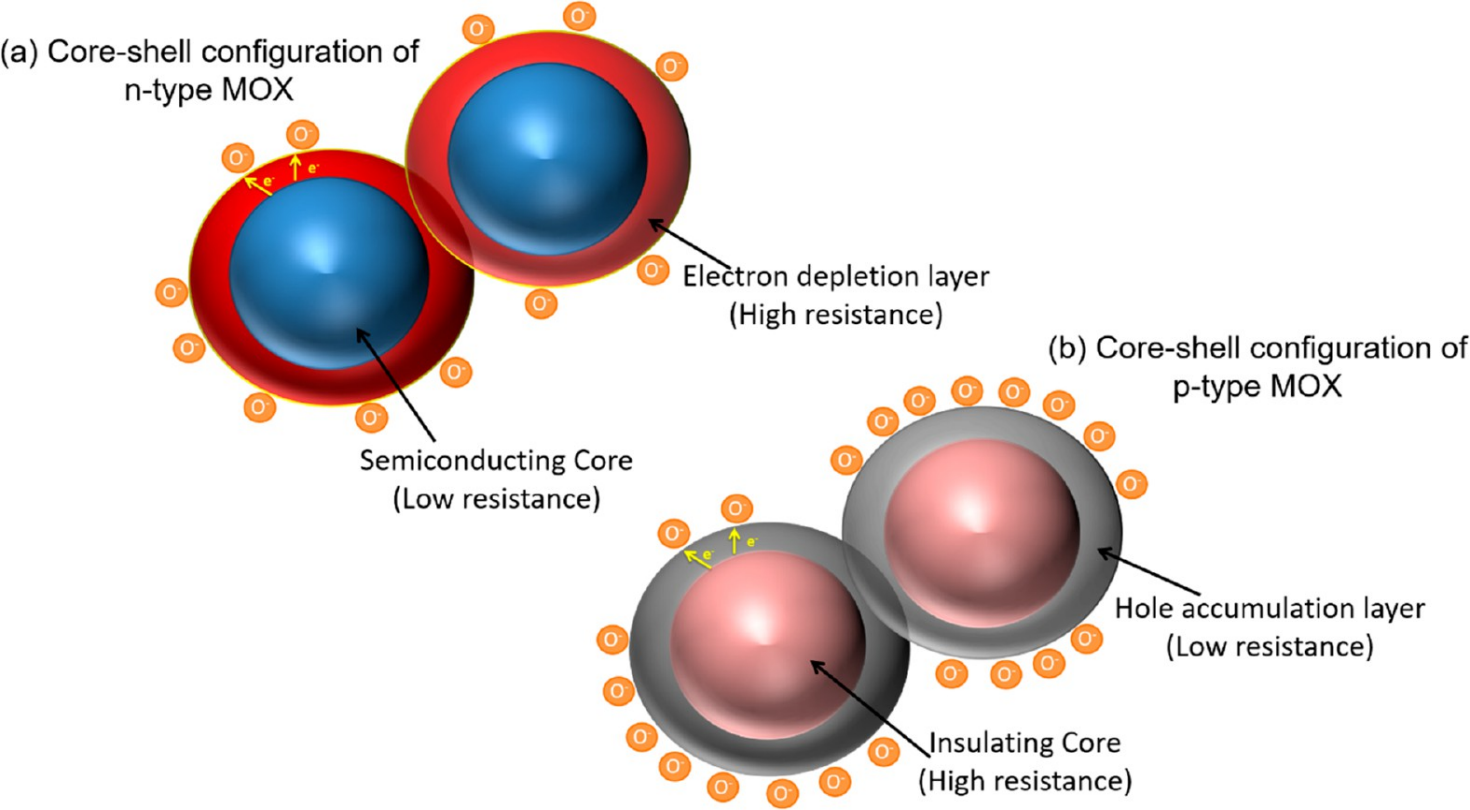
Schematic of formation of electronic core–shell structures in (a) n-type and (b) p-type metal oxide semiconductors. Redrawn from ref (39).

In order to understand the gas-sensing mechanism of n- and p-type semiconductor MOXs, it is important to understand the origin of their semiconducting nature. It is well known that, in Si-based semiconductors, the majority charge carriers (electrons or holes) can be manipulated by doping with donor or acceptors impurities.^38^ However, in wide-band-gap MOX, the nonstoichiometry determines the majority charge carriers if no intentional doping has been introduced.^39,40^ For example, the NiO p-type semiconducting character can be explained by the deficiency in metal ions, while SnO_2_ n-type behavior is a result of the generation of free electrons, as oxygen vacancies are formed.^39^ In semiconductor MOX-based chemical/gas sensors, the high response toward specific molecules can be achieved by operating the sensors at a particular optimal temperature that changes the concentration of a majority of the charge carriers and the chemical interaction with the surrounding atmosphere. Furthermore, at elevated temperatures (100–500 °C), oxygen molecules are chemisorbed and ionized (O_2_^–^, O^–^, and O^2–^) by capturing the electrons from the semiconductor surface.^41^ The chemisorption of oxygen ions that occurs on the MOX surface at different temperatures is shown in the following equations:^42^
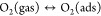
1
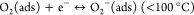
2
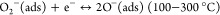
3
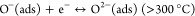
4Thus, at temperatures of less than 100 °C, adsorbed oxygen ions capture electrons from the MOX surface and become O_2_^–^, while in the temperature range of 100–300 °C, O_2_^–^ captures electrons from the MOX surface and becomes O^–^. Finally, at temperatures greater than 300 °C, O^2–^ exists on the surface of MOX. This adsorption of oxygen ions leads to the formation of an electron core–core–shell configuration. In n-type MOX such as SnO_2_, due to oxygen ion sorption, an electron depletion layer (EDL) and a shell around the semiconducting core are formed, while in p-type MOX a hole accumulation layer (HAL) and a shell near the surface and around the insulating core are formed. The core–shell configuration of both n- and p-type semiconductors is shown in Figure 1.^39^

**Figure 2:**
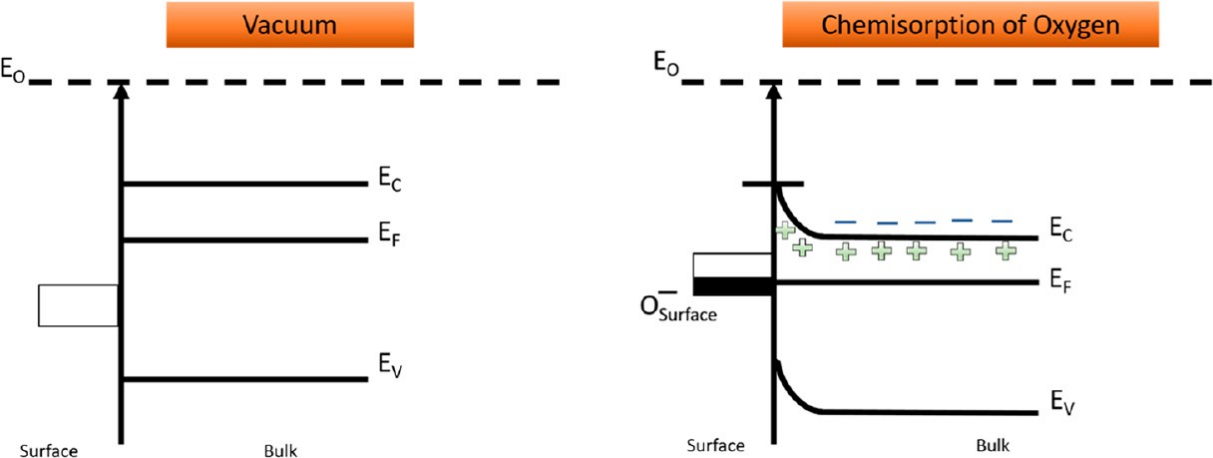
Band-bending phenomenon in n-type metal oxides due to the chemisorption of oxygen. E_O_, E_C_, E_V_, and E_F_ represent the vacuum level, conduction band, valence band, and Fermi level energies of the semiconductor.

In order to further elaborate on the formation of the EDL layer in n-type MOX, a band diagram before and after the chemisorption of oxygen is presented in Figure 2.^40,43^ In vacuum, the bands are flat and the surface states are completely empty. However, when MOX is exposed to air, oxygen chemisorption occurs on their surface. These chemisorbed ions capture electrons from the conduction band of MOX and induce the band-bending phenomenon (upward band bending). The captured electrons trapped on the surface of MOX in the form of negative ions and the EDL on n-type metal oxides is formed.^43^ On the other hand, the chemisorption of oxygen on p-type metal oxides forms the HAL.

Interestingly, Iwamoto et al.^44^ have found that the numbers of oxygen ions adsorbed on the surface of n- and p-type semiconductors are considerably different. The total amount of oxygen desorbed below 560 °C (*V*_560_) for 16 MOX was measured through the use of temperature-programmed desorption (TPD). The experiments proved that a large number of adsorbed oxygen species (large value of *V*_560_) exist on p-type metal oxides (MnO_2_, NiO, Co_3_O_4_, and Cr_2_O_3_) in comparison to the n-type ones (SnO_2_, TiO_2_, ZnO, and Fe_2_O_3_). However, among the n-type MOX, transition MOX (i.e., Fe_2_O_3_) had the highest *V*_560_ value since their oxidation state may vary. Thus, the low stability of transition-metal oxides (NiO, Co_3_O_4_, MnO_2_, and Cr_2_O_3_) is the basic reason behind the greater ionosorption of oxygen.^44^

By taking into account the core–shell configuration of MOX, the resistance of n-type MOX-based chemical/gas sensors is determined by the resistive contacts between shells (shell-to-shell contacts).^39^ If we define *R*_core_ and *R*_shell_ as the resistances of a semiconducting core and a resistive interparticle shell, respectively, then the total resistance of the n-type MOX gas sensor will be the series combination of both of these, while in the p-type MOX sensors, the conduction can be explained by the parallel path created between the wide resistive core (*R*_core_) and narrow conducting shell (*R*_shell_).^40^ Therefore, when n-type MOXs are exposed to a reducing gas such as carbon monoxide (CO), electrons are transferred to the semiconductor and CO is oxidized by the reaction with the surface-adsorbed oxygen ions. This results in a decrease in sensor resistance (increase in conductance) which is proportional to the gas analyte concentration and can be described by the following equations:
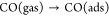
5
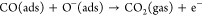
6The overall effect is a change in the density of adsorbed oxygen ions that is detected as a decrease in sensor resistance. On the other hand, the interaction of n-type metal oxides with a strong electronegative gas such as NO_2_ (oxidizing gas) increases the sensor resistance as^45^
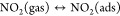
7
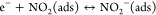
8The electron injection into a p-type MOX by the interaction with a reducing gas (CO) decreases the hole concentration in the HAL layer, thus increasing the sensor resistance. According to the literature, n-type MOX sensors generally have a higher response (measured as the change in the sensor resistance/conductance when exposed to the chemical compound relative to its value in air) as compared to p-type MOX.^46^ This is the basic reason that p-type MOXs have been less-frequently investigated as compared to the n-type ones. It is always a challenge to fabricate highly sensitive p-type MOX chemical/gas sensors. The conduction in p-type semiconductors occurs mainly in the narrow HAL, and the hole–electron recombination (electrons donated by the reducing gas) does not affect the overall resistance of the sensor significantly.^39^ Hence, the response of p-type MOX sensors is usually lower than that of n-type MOX sensors. If *S*_p_ and *S*_n_ are the responses of p- and n-type MOX sensors for particular gas analytes and semiconductor materials with the same morphology, then according to Hubner et al.^47^ they can be related by the following equation:
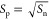
9Hence according to the above equation, it is very difficult to develop a highly sensitive gas sensor based on p-type MOX.

However, p-type metal oxides are still considered to be potential materials for new chemoresistive gas sensors due to their unique physical/chemical properties. Moreover, due to the advance in the field of nanotechnology, continuous effort has been expended in the development of p-type gas sensors. For example, sensor NiO nanowires were grown for the first time using the VLS mechanism, and they were integrated into conductometric chemical sensors that exhibited remarkable performance toward H_2_.^48^

To determine the final sensing performances, apart from the chemical reaction and conduction mechanism mentioned above, other important parameters such as the surface morphology, nanocrystalline properties, and the large surface-to-volume ratio have to be taken into account. For this reason, it is very challenging to predict the sensing behavior of an active material without experimental tests. However, in order to understand the gas-sensing mechanism of metal oxides, in situ operando experiments such as X-ray absorption spectroscopy have been performed in the past.^17,49,50^

### Device Structure and Working Principle of a Conductometric Sensor

The three main components of a conductometric gas sensor device are the following:

1. An active sensing material that must be deposited on the substrate;
2. Electrodes for the functional measurement; and
3. A heater that keep the sensor at the desired working temperature

**Figure 3:**
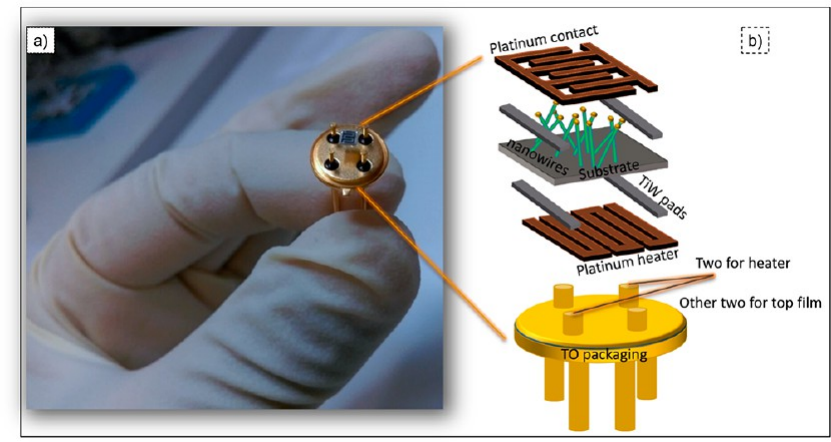
(a) Picture of the real gas-sensing device used in the Sensor Laboratory (UniBs). (b) Step-by-step fabrication of a conductometric device.

These three main components are considered while making conductometric sensing devices. However, the simplest form of a conductometric gas-sensing device can be an active sensing material (thin film, nanowires, and nanobelts) deposited between the two metal electrodes. Thanks to advances in micro/nanofabrication, different structures and dimensions can be achieved. At the Sensor Laboratory, sensing devices were prepared via dc magnetron sputtering having dimensions that were as small as 2 × 2 mm^2^.^48^ The step-by-step fabrication of a conductometric sensor and the picture of a complete sensing device are shown in Figure 3.

In particular, the first step involves the growth of nanowires on insulating substrates, such as aluminum oxide, using different growth techniques. For the electrode deposition, first an alloy of Ti/W and Pt soldering pads was deposited on the substrate using sputtering. Afterward, the interdigitated Pt electrodes were deposited on the nanowires by using shadow masking in order to have higher electrical conductance and mechanically stable gold wire bonding. The pads were used for the electro-soldering of gold wire. For temperature-dependent sensing measurements, Pt heating elements were also deposited using dc magnetron sputtering on the back side of the substrates. To achieve this, the successive deposition of Pt pads (using a Ti/W adhesion layer) and Pt contacts has been performed. Prepared devices were finally mounted on TO packages using electro-soldered gold wires (Figure 3b).

The working principle of chemoresistive gas sensors relies on the change in electrical resistance/conductance of the active sensing materials upon interaction with the gas analyte. The nature of the sensitive materials (n-type or p-type) and the target gas (oxidizing or reducing) governs the increase or decrease in electrical resistance. In the class of solid-state metal oxide-based chemical/gas sensors, conductometric ones are the most investigated and exploited devices and are designed for the detection of toxic as well as inflammable gases in the surrounding atmosphere and for monitoring technological processes.^51−53^ In gas-sensing measurements, the change in electrical conductance or resistance of a sensor upon interaction with the gas is measured at an optimal working temperature. A constant dc voltage whose value may vary depending on the resistance of the sensor is kept constant during the measurement. To find the optimal working temperature of the sensor for a particular gas analyte, the response of the sensor is recorded at different temperatures. The maximum in the response vs temperature plot for a given gas analyte is considered to be the optimal working temperature. The main advantages of metal oxide conductometric sensors are low production cost, ease of fabrication, and simple operation thanks to their intrinsic properties as well as manufacturing techniques.^54^ This implies that mass production at reasonable cost is possible using well-engineered metal oxide conductometric sensors. Brief details of the conductometric gas-sensing measurement setup developed and used in the Sensor Laboratory are described in the Supporting Information.

### Important Sensing Parameters

**Figure 4:**
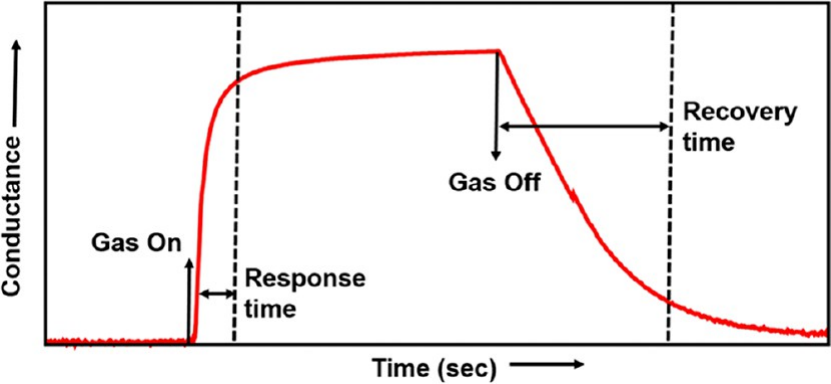
Typical response curve of a conductometric gas sensor.

**Figure 5:**
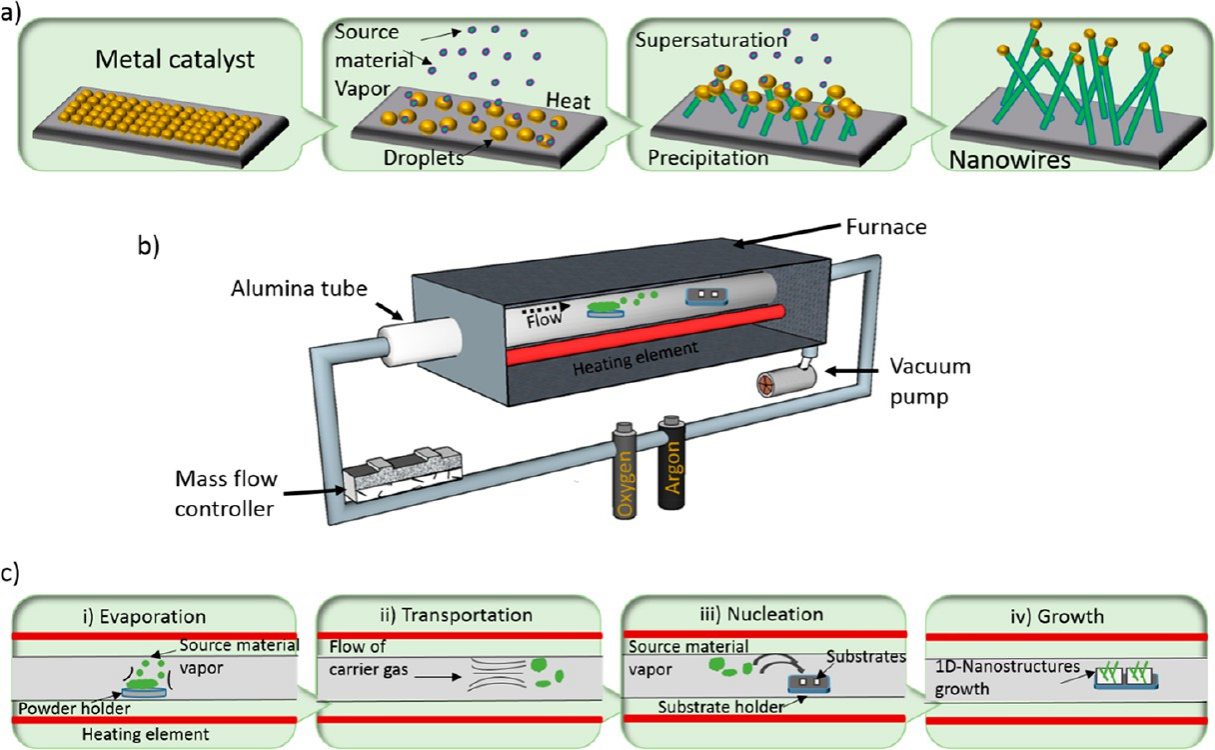
(a) VLS mechanism for the growth of nanowires. (b) Schematics of the custom tubular furnace used for the fabrication of MOX nanowires. (c) Steps involved in the growth process of nanowires.

In general, the electrical conductance/resistance of the metal oxide-based conductometric gas sensors changes with exposure to the target gas. The nature of the sensitive materials (n-type or p-type metal oxide semiconductors) and the target gas (oxidizing or reducing) governs the electrical resistance increase or decrease. For example, when an n-type semiconductor is exposed to a reducing gas, the resistance decreases, whereas upon exposure to an oxidizing gas, the resistance increases. Figure 5 reports the typical dynamic response curve of a gas sensor. According to this curve, a change in conductance or resistance is registered when it is exposed to a gas analyte (“gas on”). After reaching a stable conductance value in the presence of the gas, the air flow is restored (“gas off”) and the sensor recovers to its initial value (baseline conductance and resistance). In Figure 4, the response and recovery times of sensors can be determined.

Some of the important sensing parameters that are used to characterize the sensing performances are the following:^55^

1. Sensitivity (S): Sensitivity can be defined as the change in the measured signal per analyte concentration unit (i.e., the slope of a calibration curve). It should be noted that the calibration curve is a log–log graph between the sensor response and the analyte concentration.
2. Selectivity: Selectivity determines whether a sensor can respond selectively to a group of analytes or even specifically to a single analyte. It generally expressed as

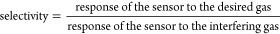
10Thus, according to the above equation, selectivity is the ability of the sensor to discriminate the response for a particular gas analyte from other interfering gases.

1. Response time: This is defined as the time required for a sensing device to respond to a step concentration change from zero to a certain concentration value. In addition, the response time can be calculated as the time required for a sensor resistance/conductance to reach 90% of the equilibrium value during exposure to a gas analyte. The response time indicates how fast or slow a sensor can respond when a gas analyte is introduced into the test chamber.
2. Recovery time: When the step concentration changes from a certain value to zero, the recovery time is the time taken by the sensor to return to its initial signal value. In addition, the recovery time can be calculated as the time required for the sensor resistance/conductance to return to 70% of its original value of resistance/conductance in air. The recovery time indicates how quickly or slowly the sensor recovers to its initial conductance when the air flow is restored.
3. Stability: This defines the ability of a sensor to provide reproducible results for a certain period of time.
4. Detection limit: This can be defined as the lowest concentration of gas analyte detected by the sensor under experimental conditions such as temperature. The detection limit can be calculated by fitting the log–log plot of response vs different gas concentrations (calibration curve). It is the minimum concentration that a sensing device can detect under optimized conditions by giving a detectable signal.
5. Optimal working temperature: This corresponds to the temperature at which the sensor gives the maximum response to the gas analyte.

All of the above-mentioned parameters are used to characterize the sensor performance. An ideal sensor should be highly sensitive, selective, and stable with a low detection limit and a short response time. However, it is very difficult to optimize all of these parameters together in a single sensing device, and researchers usually make an effort to approach only some of them.

## Fabrication of Metal Oxide-Based One-Dimensional Nanostructures and Their Characteristics

For 1D nanostructure fabrication, the most important requirements are dimensions, morphology control, crystalline properties, and uniformity.^23,41^ Furthermore, a preferential growth direction with a faster growth rate and higher yield is mandatory.

One-dimensional nanostructures can be prepared by following two different technologies: bottom-up and top-down.^56^ Top-down technology is based on standard microfabrication equipment with the deposition and etching of planar structures to reduce their lateral dimensions down to the nanoscale level. Other techniques such as focused ion beams, electron beams, and X-ray lithography have been used.^57−59^ The major hindrances that come with top-down approaches are the long preparation time and remarkably elevated costs. However, one should not forget the positive aspects of this approach, such as using a well-known technology coming from the semiconductor industry. In addition, the structures can be prepared directly on planar substrates, which allows for easier successive electrical connection with the macro world. In the literature, many reports have been published on the fabrication of 1D nanostructures with a top-down approach.^60−63^

On the contrary, bottom-up synthesis is based on the assembly of molecular building blocks or chemical synthesis directly into nanosized morphology. This approach presents many advantages such as high purity, crystallinity, and the easy achievement of reduced dimensions of the fabricated materials, in addition to the low cost of the experimental setups together with the possibility to easily vary the intentional doping and formation of junction-based devices.^19,64^ However, the major issue regards the integration into planar substrates necessary to take full advantage of the useful properties. Furthermore, nanostructure patterning and alignment may be difficult.

Thus, bringing together both the bottom-up (for the fabrication of the nanostructures) and top-down (for large scale fabrication) approaches is the most promising scheme for producing highly functional materials.

To grow MOX nanowires, various techniques such as the hydrothermal method,^65^ sol–gel chemistry,^66^ and electrospinning^67^ have been proposed and successfully implemented by the researchers for gas-sensing applications. However, in this review we will mainly focus on the bottom-up growth techniques (i.e., vapor/liquid phase growth methods and thermal oxidation that were used in the Sensor Laboratory).

### Vapor-Phase Growth

Vapor-phase growth was proposed by Wagner and Ellis in the early 1960s and is one of the first techniques developed for preparing micro- and nanostructures.^68^ It consists of the evaporation of source material in a tubular furnace (Figure 5b). The evaporated source material is transported by a carrier gas from a hotter region to a colder one. Here the material condenses and nucleates on growth sites (Figure 5a). The actual idea was presented for silicon whiskers and afterward was adapted for other materials such as metal oxides. Condensation on the substrate could occur according to two different mechanisms: vapor–solid (VS) and vapor–liquid–solid (VLS).

The VS growth takes place when the NW crystallization emerges from the direct condensation of the source material without the presence of any catalyst.^69^ In this mechanism, source materials are heated under high temperature from the vapors and directly condensed on the target substrates placed in the relatively low temperature region. Once the condensation process starts, the initially condensed molecules create seed crystals that serve as nucleation sites for the further growth of nanowires.^70^

On the other hand, the VLS mechanism^71,72^ is named after the three different phases of material involved in the growth process: the vapor state of the source material, a liquid catalyst droplet, and the solid crystalline nanostructure that is produced. Catalysts can be deposited on the substrates using different techniques such as colloidal solutions and magnetron sputtering. Liquid metal clusters or catalysts act as favorite adsorption sites for the vapor, and when supersaturation is reached, there is a segregation normally at the bottom of the cluster and the nanowire growth starts. Metal catalysts are essential in the VLS mechanism, but not all metals can be effective. These catalysts need to fulfill some requirements, such as they must be liquid at the deposition temperature, they must be inert to chemical reactions, the vapor pressure of the catalyst component over the liquid alloy should be small, and they must not make intermediate solids.^73−75^

The most widely used catalysts are noble metals such as gold, ruthenium, platinum, and palladium.^75^ The dimensions of the nanowires can be directly related to the size of the catalyst clusters.^41,76^ This can be either by matching the size or by the process involving the catalyst curvature in which lattice matching and strain matching are important.^75^

One-dimensional nanostructure growth via the VLS or VS mechanism can be achieved using a tubular furnace that is able to reach the high temperatures necessary for material evaporation.^19,77,78^ In order to reduce and control the pressure inside the alumina tube, the system must be connected to vacuum pumps. This will reduce the evaporation temperature and avoid the inclusions of unintentional dopants into the growing nanostructures. In order to ease the mass transport, a gas carrier is normally used, and mass flow controllers inject the carrier gases (normally argon) inside the system (Figure 5b). For better reproducibility, a homemade NI LabVIEW virtual instrument (VI) that fully controls the system has been used to control the whole process. MOX powder is placed in an alumina boat, in the middle of the alumina tube, at a temperature which is high enough to induce the evaporation of source material. The growth substrates (with and without catalyst) are placed on the alumina substrate holder in a colder region of the tube. During the heating step, the flow of the carrier gas is restricted to the reverse direction, from the substrates to the powder, in order to avoid any undesired condensation of the powder under undesired growth conditions. As the furnace reaches the desired temperature, the flow is switched to the direct direction, from powder to the substrates, and the deposition begins. The deposition time depends on the different growth parameters such as the amount of material desired, the preferred morphology, and so on. During cooling, the flow is kept in the reverse direction (Figure 5c). It is possible to tune the achieved morphology by changing the pressure inside the alumina tube, the condensation temperature, the carrier gas flow, the deposition time, and the catalyst used for the fabrication process.

Different n-type ZnO,^79−82^ SnO_2_,^83−86^ In_2_O_3_,^87−89^ and WO_3_^77^ and p-type NiO^48,78,90^ metal oxide nanowires deposited on various substrates were successfully used in the Sensor Laboratory with this experimental setup. Some major advantages and disadvantages related to the vapor-phase growth technique in tabular form are presented in the Supporting Information.

### Structural and Morphological Characterization

Tin dioxide (SnO_2_) is a MOX that has been extensively exploited for gas-sensing application in many different forms such as thick films, thin films, and nanowires.^83,84,91,92^ Our group^28^ was the first to present a report on the synthesis of tin dioxide nanobelts using the simple catalyst-assisted evaporation–condensation of source material (in powder form) for gas-sensing application. This work encouraged many researchers worldwide to work in the field of nanostructured MOX synthesis for gas-sensing application. Furthermore, in 2007 our group^85^ reported on the conductometric gas-sensing application of tin dioxide nanowires grown by VLS and VS mechanisms. For VLS growth, platinum (Pt) is used as the catalyst to promote the nucleation of nanowire growth. The evaporation of source material (SnO_2_) was performed at 1370 °C and the substrate were placed at 470 °C to achieve nanowire growth by VLS, while for catalyst free growth (VS), the substrates were placed at 330 °C. The higher temperature permits the formation of melted Pt–Sn clusters, which aids nanowire nucleation. HR-TEM reveals that the growth of nanowires proceeds along a specific crystallographic direction (i.e., [100]). No evidence of the epitaxial relationship between the nanowires and the substrate was established in the case of VS growth. Instead, in the VLS process, the presence of the Pt catalyst was attributed to the promotion and formation of aligned nanowires.

Following this interesting work, many other works on the synthesis of SnO_2_ nanowires using the same technique but optimizing the growth condition for different substrates and catalysts have been reported.^83,86,93,94^ Gold (Au) is another optimal catalyst for the synthesis of SnO_2_ nanowires with a diameter of 100 nm to 1 μm.^93^ Interestingly, throughout the literature many different catalysts (Au, Pt, Ag, and Sn)^86,94,95^ were used in order to obtain NW morphology for chemical/gas sensing, which also confirms that SnO_2_ is one of the profoundly exploited materials.

**Figure 6:**
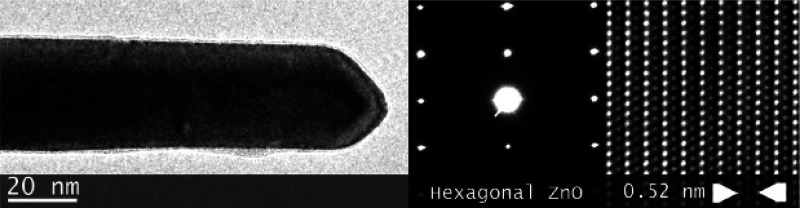
(Left) TEM bright-field image, (center) electron diffraction pattern of the ZnO nanowire, and (right) high-resolution image of the crystalline arrangement. Adapted with permission from ref (82). Copyright (2007) IOPscience.

Figure 6 shows the high-resolution images and crystalline pattern of a ZnO nanowire grown by the VS mechanism on an alumina substrate.^82^ The nanowire features a diameter ranging between 72 and 78 nm and appears uniform with a tapered termination, likely to be determined by the crystalline aspect. The nanowire grown by this method exhibits single-crystal growth.

A number of reports has been published in the past decade on ZnO nanowire synthesis using the VLS mechanism to study the influence of substrate temperatures and catalysts.^79,96,97^ The most prominent catalysts are noble metals such as Au and Pt. However, the use of different types of substrates, such as crystalline instead of polycrystalline substrates, influences the ZnO nanowire growth.^98^ In this particular case, the nanowires process a diameter ranging from 50 to 100 nm with a random orientation, along with some planar structures such as platelets that were also observed. However, the nanowires were found to be single-crystalline in nature. According to the literature, it has been observed that the ZnO nanowires grown using the vapor-phase technique are very sensitive to the substrate type and temperature.^79,96^

**Figure 7:**
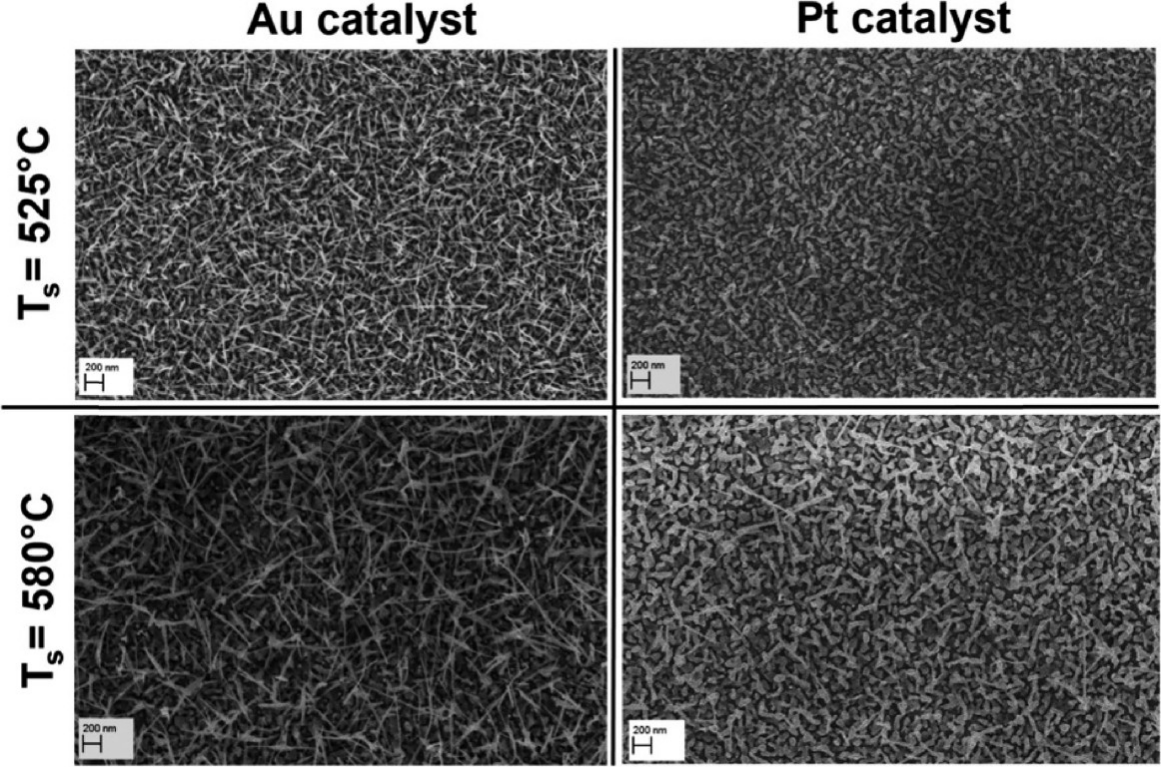
SEM images of WO_3_ nanowires at an evaporation temperature of 1100 °C, a pressure of 1 mbar, and an argon flow of 100 sccm for Au and Pt catalysts at substrate temperatures of 525 and 580 °C, respectively. Adapted with permission from ref (77). Copyright (2019) American Chemical Society.

In a recent report, the vapor-phase growth has also been successfully used for the synthesis of WO_3_ nanowires.^77^ In the case of WO_3_, this method has been rarely used.^99^ It has been observed that the evaporation and substrates temperatures for the nanowire growth are lower compared to the ones used for other n-type semiconductor materials (SnO_2_ and In_2_O_3_). The influence of different catalysts (Au and Pt), substrate temperatures, and deposition time was analyzed. In the case of a platinum catalyst, no growth of nanowires was observed at substrate temperatures of 525 and 580 °C. On the other hand, using Au as a catalyst, the nanowire growth is uniform, and when the substrate temperature was increased to 580 °C, the nanowires grow longer as shown in Figure 7. Their diameter was found to be in the range of 10–30 nm with a length of less than 100 nm.

On the other hand, p-type metal oxides have been the least studied using this growth technique. Kaur et al.^48^ at the Sensor Laboratory were the first to report NiO nanowires synthesis using VLS for the integration into gas-sensing devices. The required evaporation temperature and the optimal growth temperature were higher with respect to the previously reported n-type semiconducting materials. For the first time, the authors presented details of the growth, including the effect of different experimental parameters such as the catalyst (Au, Pt, or Pd), substrate temperature, and different carrier gas flows. The achieved nanowires were uniform and very dense using Au as a catalyst with an Ar flow of 100 sccm and 930 °C as the condensation temperature. The deposited nanowires were long and thin and had a denser morphology with diameters ranging from 16 to 50 nm and were single-crystalline in nature using Au in comparison to other catalysts.

### Thermal Oxidation

Thermal oxidation is among the easiest and highest-yielding techniques for growing different metal oxide nanostructures.^100−102^ This technique has several advantages such as the production of highly crystalline materials, easy patterning, scalability for a large amount of production with high yields, and possible operation at atmospheric pressure for some materials.^5^ However, the major drawback is the time required for the growth process, which can be hours.

**Figure 8:**
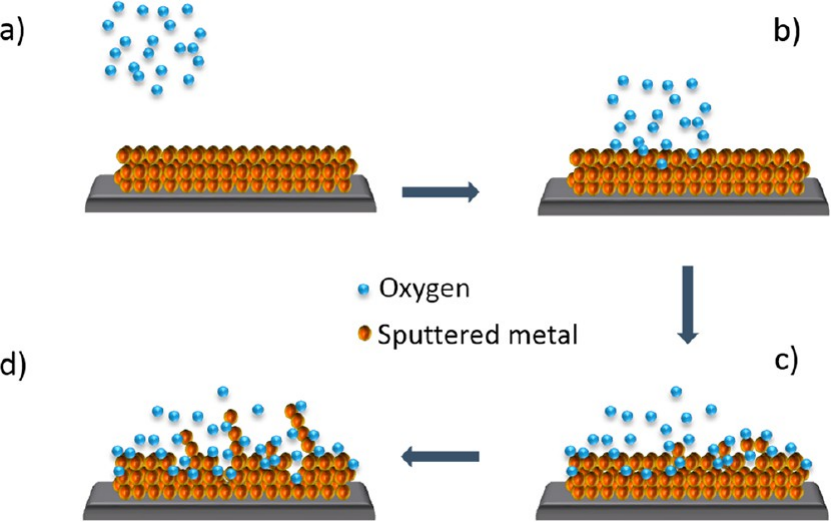
Growth mechanism of thermal oxidation. (a) Presence of oxygen ions in the atmosphere. (b) Surface reactions between oxygen ions and metallic atoms. (c) Diffusion of oxygen into the metal and nucleation of nanowires. (d) Growth of nanowires and oxidation.

For some specific metal oxides such CuO, the growth mechanism is fully understood. Zappa et al. have described that during the formation of thermally oxidized CuO NWs, first the metallic Cu oxidized to form a Cu_2_O film (Cu + O_2_ → Cu_2_O). This Cu_2_O film further oxidized to CuO (Cu_2_ O + O_2_ → CuO) and produced CuO NWs. Moreover, Arafat et al.^100^ have investigated the effect of mechanical stress on thermally oxidized TiO_2_ nanowire growth. Indeed, the induced stress has significantly improved the nanowire coverage and confinement. Due to the high thermal energy, the surface reactions are occurring with oxygen atoms present in the atmosphere (Figure 8a,b).^103^ The process of nucleation starts due to oxygen atom diffusion inside the metal layer because of the mechanical stress. Furthermore, the nanowire growth is also promoted by the presence of in-phase tensile stresses (Figure 8c), and the uppermost layer is highly defective, porous, and oxidized. For further oxidation, metal atoms find two pathways, lattice or grain-boundary diffusion to reach the interface of oxide/air. Due to the lattice diffusion, the oxide layer thickness increases, while the diffusion at grain boundaries results in nanowire formation (Figure 8d).^103−105^

The growth of nanostructures consists of two steps: the deposition of a metallic layer followed by the thermal oxidation shown in Figure 8. For the metal layer deposition, different techniques can be applied such as electrodeposition, magnetron sputtering, and thermal evaporation. The substrate with the patterned metal deposition must undergo thermal oxidation treatment; therefore, it is placed in the tubular furnace in an oxidizing atmosphere (a mixture of oxygen and argon). Controlling different parameters such as the furnace temperature, deposition time, atmospheric composition, and gas flow allow the control of nanostructure morphology and uniformity. Using this technique, ZnO,^105^ WO_3_,^30,106^ Nb-doped WO_3_,^107,108^ and CuO^109^ nanowires have been prepared for the chemical sensing application.

Some major advantages and disadvantages related to the thermal oxidation technique in tabular form are presented in the Supporting Information.

### Structural and Morphological Characterization

**Figure 9:**
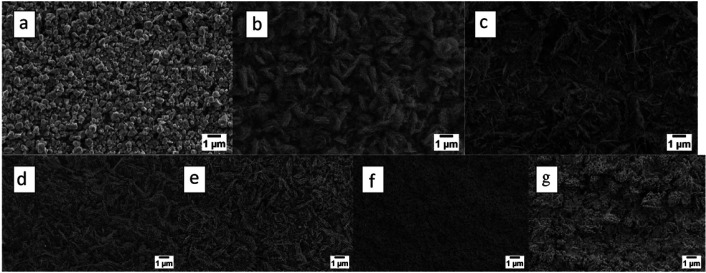
Influence of oxidation temperature: (a) 200, (b) 300, (c) 400, (d) 500, (e) 600, (f) 700, and (g) 800 °C. The atmospheric composition was 100% O_2_, while the metallic zinc sputtering time was 3 h (4.5 μm) at RT. All images are at 20K magnification. Adapted with permission from ref (105). Copyright (2013) IOPscience.

Assorted metal oxide-based nanowires grown by using thermal oxidation techniques are presented in the literature. Adopting this technique also provides the flexibility to fabricate the nanowires directly onto the active transducer. ZnO is one of the most exploited materials in different applications.^80,110,111^ Using thermal oxidation, highly scalable ZnO nanowires have been prepared directly on alumina substrate.^105^ The morphology largely depends on the oxidation temperature and oxidation time. Figure 9 shows the temperature-dependent morphology of ZnO nanostructures grown using thermal oxidation. Clearly, between 400 and 600 °C, nanowire morphology was observed under a controlled atmosphere of oxygen and argon, while at 200 °C, ZnO nanoparticles are formed.

Larger nanoparticles are obtained at 700 and 800 °C with a porous-like structure. However, ZnO nanostructures obtained by thermal oxidation are less well defined in comparison with the ones prepared by VLS. Furthermore, the oxygen composition strongly affects the morphology. At 400 °C, the decreasing oxygen ratio at atmospheric composition promotes the formation of dense and longer nanowires, although the same composition has no effect on the morphology at lower temperatures.^105^

**Figure 10:**
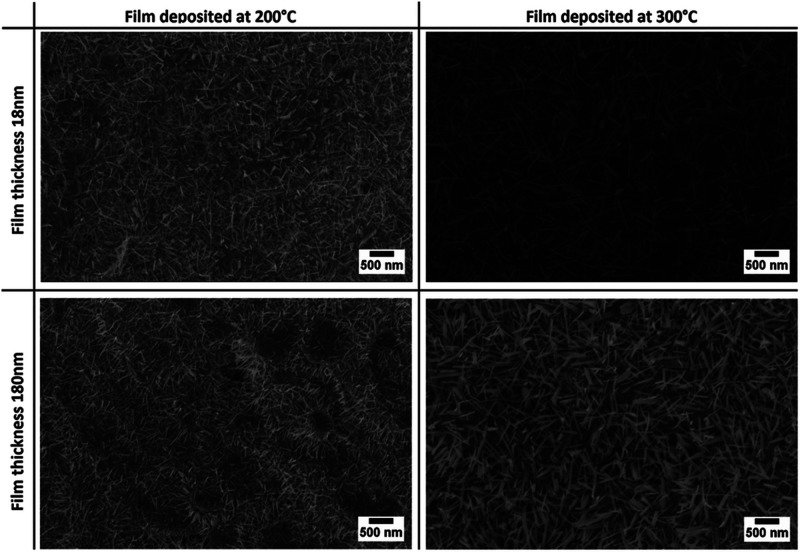
SEM pictures of oxidized 18 and 180 nm tungsten films deposited at different temperatures by rf magnetron sputtering. Adapted with permission from ref (106). Copyright (2014) Royal Society of Chemistry.

In the case of WO_3_ nanowire growth by thermal oxidation, the thickness of the metallic film and the temperature play fundamental roles.^106^ This report presents two different metallic tungsten layer thicknesses (18 and 180 nm) and film deposition temperatures (200 and 300 °C) via thermal oxidation in the tubular furnace at 550 °C for 1 h with an oxygen flow of 2 sccm. The morphological investigation shows that the sputtering temperature seems to have a strong influence on the nanowire size. The nanowires have a larger diameter and a longer length for a layer produced at 300 °C than for one produced at 200 °C; however, the average diameter in both cases is 40 nm as shown in Figure 10. The influence is more prominent in the case of the metallic layer prepared at 300 °C rather than 200 °C, which is due to the abundance of bulk tungsten metal present underneath nanowires (confirmed from the GI-XRD study). However, after the thermal treatment at temperatures of around 400 °C, this metal layer is also oxidized.

In a recent article, a successful attempt to grow Nb-doped tungsten oxide nanowires by thermal oxidation has been reported.^107^ To achieve Nb-WO_3_ nanostructures, the metal layer deposition was performed with magnetron sputtering using a target of W with a variable number of niobium insets. An alloy of metallic tungsten–niobium was deposited on an alumina substrate, with a composition that varies with the number of Nb insets. With only four stubs inserted, the presence of Nb was 3 atom %. Increasing the number of stubs to 12, about 9.5 atom % was achieved. Furthermore, the functional analyses showed that Nb-WO_3_ nanowires have an excellent hydrogen-sensing capability compared to that of pristine ones. This new and simple perspective of doping nanostructures by the thermal oxidation technique opens a new possibility to change the sensing response of these nanostructured chemical/gas sensors.

Thermal oxidation used for p-type semiconducting material CuO shows a complex process for the nanostructures’ growth. A metallic copper layer (from 300 nm to 3 μm) was deposited by rf magnetron sputtering on alumina substrates at RT and 200, 300, and 400 °C.^109,112^ Copper is a very reactive metal. It reacts with oxygen in the ambient atmosphere and creates a thin layer of copper oxide. This thin layer is pernicious to nanowire growth; therefore, it is essential to remove this layer before thermal oxidation. Techniques such as wet chemical etching and plasma etching can be used to remove this layer.

**Figure 11:**
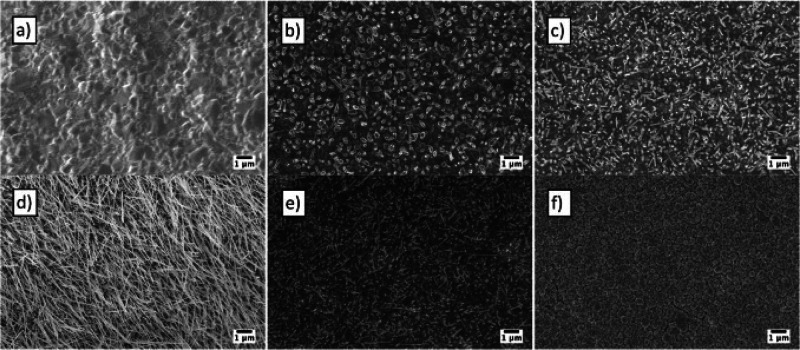
Influence of oxidation temperature: (a) 600, (b) 500, (c) 400, (d) 300, (e) 250, and (f) 200 °C. The atmospheric composition was 80% O_2_ and 20% Ar, while the sputtering time was 3 h at RT (1.8 μm). All images are at 20K magnification. Adapted with permission from ref (109). Copyright (2013) Elsevier.

The complete oxidation process first involves the oxidation of metallic Cu to a Cu_2_O film, after which the Cu_2_O film is further oxidized to CuO, generating CuO nanostructures.^113^ The oxidation temperature has a huge influence on nanowire morphology and uniformity. Figure 11 shows the morphology variation as the oxidation temperature increases from 200 to 600 °C. However, the best growth temperature was found to be around 400 °C for vertically aligned nanowires and 300 °C for randomly oriented nanowires.

## Metal Oxide Nanowire Chemoresistive Gas Sensors

**Figure 12:**
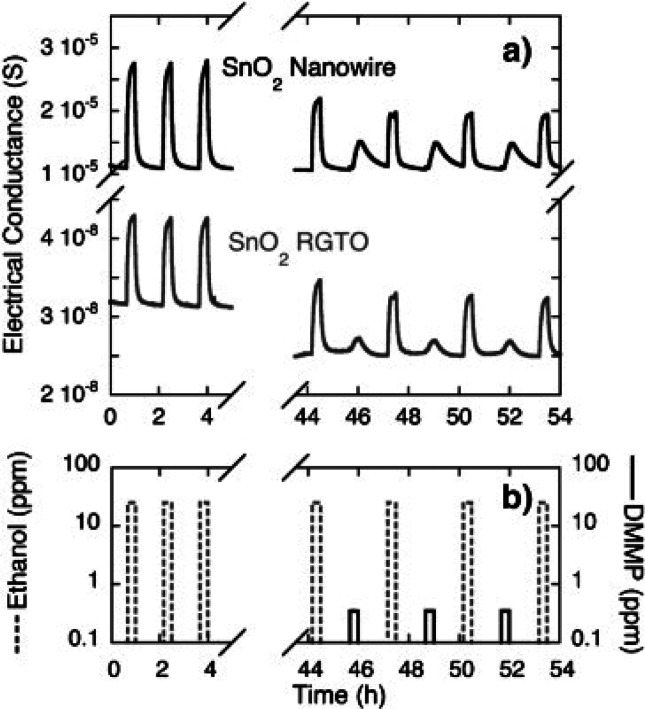
Dynamic response of SnO_2_ nanowires (black line) and RGTO (gray line) sensors to different injections of ethanol (25 ppm) and DMMP (0.2 ppm). The comparison between the ethanol sequence and the ethanol–DMMP sequence shows the poisoning effects due to DMMP exposure, also at weak concentrations. Adapted with permission from ref (83). Copyright (2009) Elsevier.

As discussed previously, our group^85^ has reported the growth of uniform single-crystalline SnO_2_ nanowires using the VLS mechanism on a polycrystalline alumina substrate. This report serves as a benchmark for their further application in chemical sensing. Chemical sensors based on SnO_2_ nanowires for the detection of chemical warfare agents (CWAs) such as dimethyl methyl phosphonate (DMMP) were developed.^83^ SnO_2_ nanowire sensors showed superior performance (Figure 12) as compared to SnO_2_ thin films which were prepared using a rheotaxial growth and thermal oxidation (RGTO) technique. Indeed, the SnO_2_ nanowire sensors showed high sensitivity toward a lower concentration of DMMP, even lower than the respective CWAs IDLH values (immediately dangerous to life and health).

In both of the above-mentioned works,^83,85^ Pt was used as a catalyst to grow SnO_2_ nanowires. However, recently Zappa et al.^114^ have grown SnO_2_ nanowires using three different catalysts (Au, Pd, and Sn) with VLS that have been integrated into gas-sensing devices. Interestingly, at high temperatures, Pd-catalyzed SnO_2_ nanowires exhibited better performance than did Au and Sn nanowires. However, the reason behind this behavior is still not clear.

**Figure 13:**
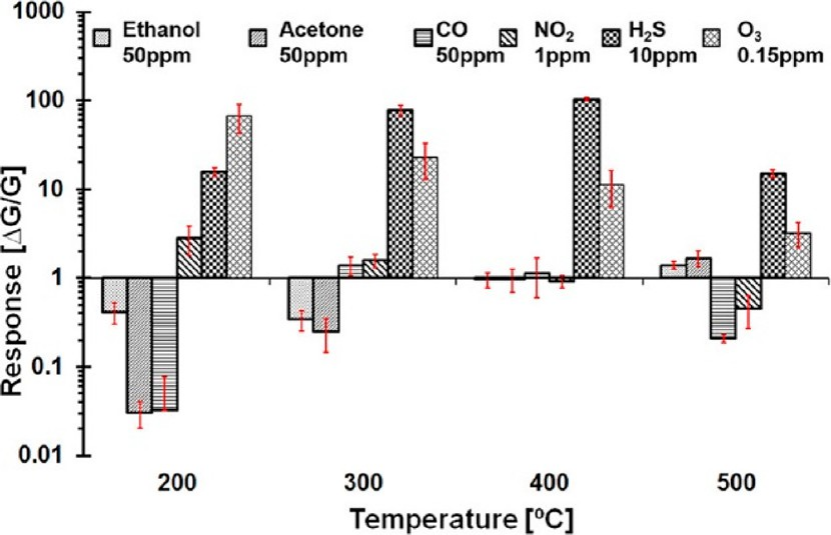
Temperature-dependent response of the WO_3_ sensing device measured with a relative humidity of 50% at 20 °C. Adapted with permission from ref (^77^). Copyright (2019) American Chemical Society.

Furthermore, WO_3_ nanowires have been grown using both the VLS mechanism and thermal oxidation for chemical sensing applications. In the VLS-grown WO_3_ nanowires, two different catalysts (i.e., Pt and Au) have been used, and the conductometric sensors based on these nanowires showed remarkable performance with respect to O_3_ and H_2_S as compared to the other interfering compounds such as ethanol and acetone (Figure 13).^77^

**Figure 14:**
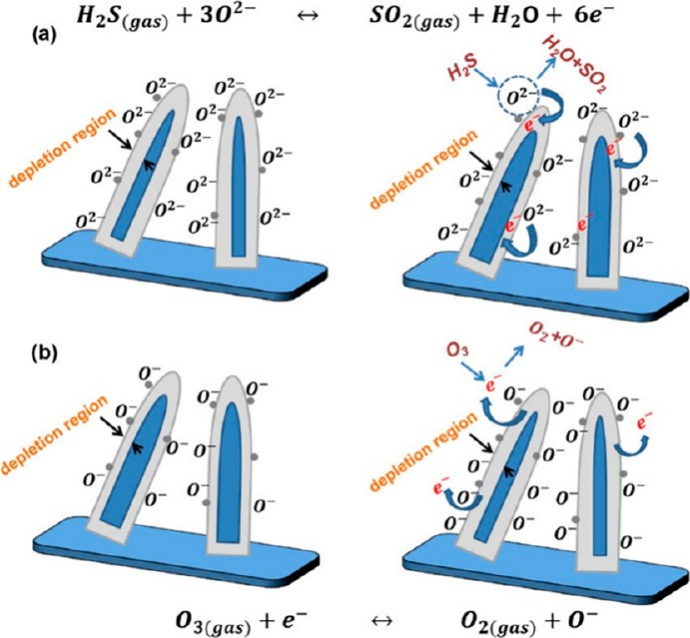
Sketch of the proposed gas-sensing mechanism of the WO_3_ nanowire sensor system toward (a) H_2_S and (b) O_3_. Adapted with permission from ref (^77^). Copyright (2019) American Chemical Society.

Kaur et al.^77^ have suggested that when H_2_S gas molecules interact with the surface-adsorbed O^2–^ ions (present on WO_3_ nanowires), extra electrons are donated to WO_3_, resulting in a decrease in the depletion layer thickness (Figure 14a) and hence an increase in the conductance of WO_3_ sensors. The whole process can be explained by the following equation
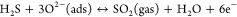
11When O_3_ gas molecules interact with the WO_3_ nanowires, due to their highly accepting character, the concentration of the surface-adsorbed O^–^ ions increases, which increases the electron depletion layer thickness (Figure 14b). Hence, the conductance of the WO_3_ sensor decreases when interacting with ozone. The whole process can be explained using the following equation
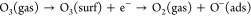
12It should be noted that the optimal operating temperatures for H_2_S and O_3_ were 400 and 200 °C, respectively, which increase the selectivity toward a specific gas by varying the sensor temperature.

**Figure 15:**
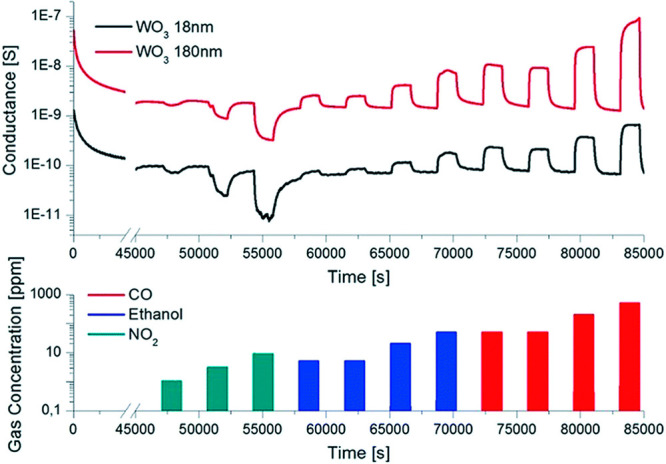
Dynamic response of the WO_3_ sensing devices toward some oxidizing (NO_2_, aquamarine color, 1–3–9 ppm) and reducing (ethanol, blue color, 5–5–20–50 ppm) (carbon monoxide, red color, 50–50–200–500 ppm) gas chemical compounds, measured at 200 °C with a relative humidity of 50% at 20 °C. Adapted with permission from ref (106). Copyright (2014) Royal Society of Chemistry.

On the other hand, WO_3_ nanowires grown using thermal oxidation^106^ showed excellent performance toward gases such as CO and NO_2_. Interestingly, in this work, the WO_3_ nanowire sensors prepared from the thermal oxidation of a 180 nm W metal film showed better performance as compared to those prepared from an 18 nm metal film (Figure 15). Zappa et al.^106^ suggested that the 180 nm metal film exhibits a higher density of nanowires compared to the 18 nm film. Hence, the increased surface area results in an enhancement of the sensing performance.

**Figure 16:**
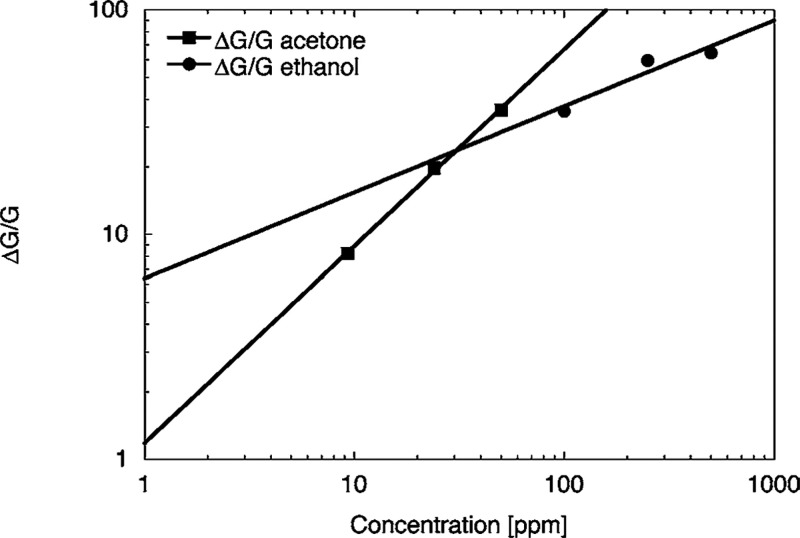
Response of zinc oxide nanowires toward acetone and ethanol as a function of the concentration at an operating temperature of 400 °C. The response follows the power behavior and reveals a very low detection limit. Adapted with permission from ref (79). Copyright (2007) Springer Nature.

Similar to WO_3_, ZnO nanowires were also prepared using both VLS growth and thermal oxidation and were integrated into chemical sensing devices. Those prepared using VLS showed excellent performance toward acetone and ethanol with a detection limit lower than 1 ppm.^79^Figure 16 shows their response as a function of acetone and ethanol concentrations.

**Figure 17:**
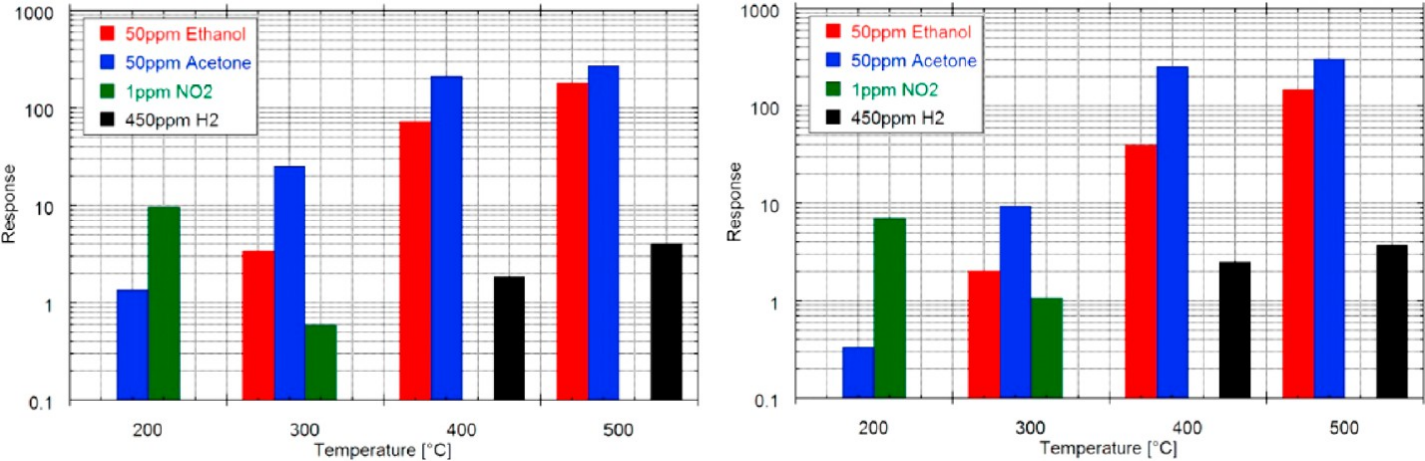
Sensitivity toward target gases at various temperatures for ZnO T400 (left) and ZnO T500 (right). The relative humidity was set at 50% at 20 °C. Adapted with permission from ref (105). Copyright (2013) IOPscience.

**Figure 18:**
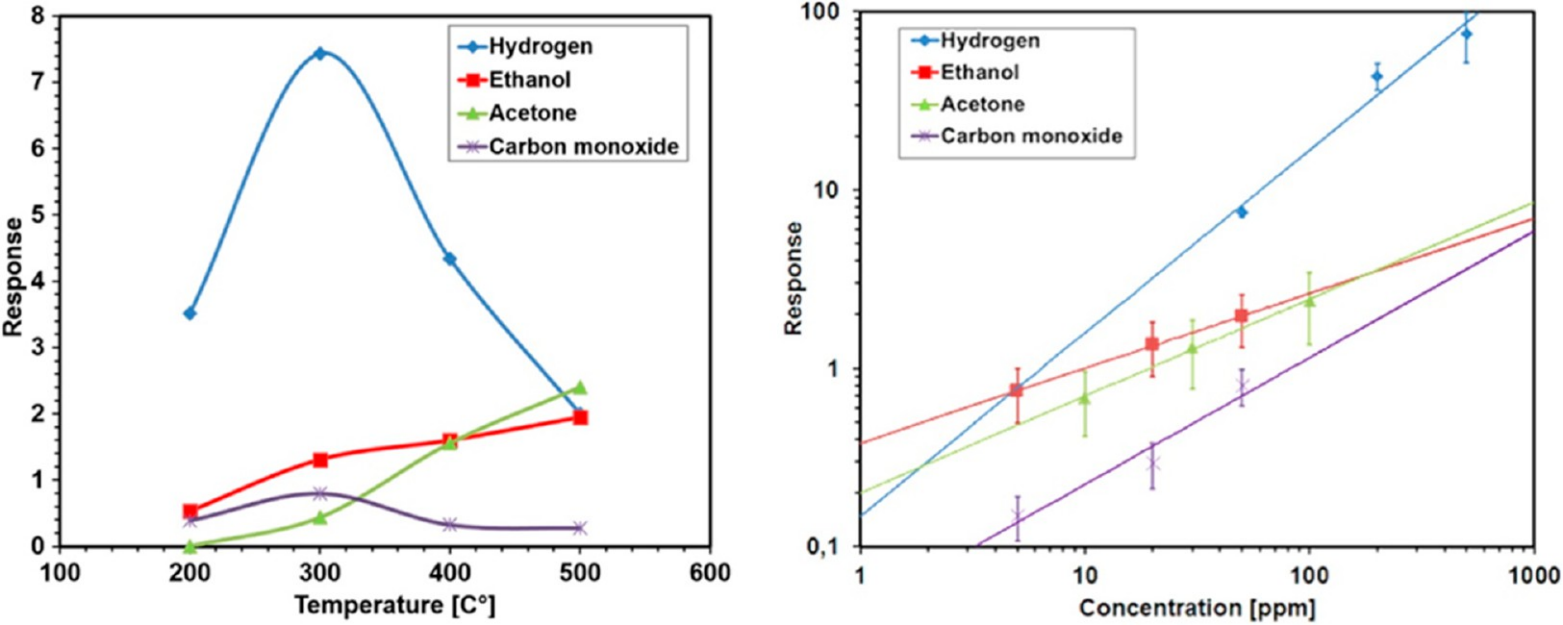
(Left) Response of NiO nanowires toward target gases: hydrogen (blue) 50 ppm, acetone (red) 100 ppm, ethanol (green) 50 ppm, and carbon monoxide (purple) 50 ppm. Relative humidity 50% at 20 °C. (Right) Calibration curves for NiO sensor devices toward hydrogen_1 V (blue) at 300 °C, acetone (green) and ethanol (red) at 500 °C, and carbon monoxide (purple) at 300 °C. Relative humidity 50% at 20 °C. Adapted with permission from ref (48). Copyright (2016) IOPscience.

The ones prepared by thermal oxidation (grown at 500 °C) showed a selective response toward NO_2_ gas at 200 °C with a detection limit of about 200 ppb.^105^ At higher temperatures (500 °C), the response to ethanol and acetone increased as compared to the response to H_2_, but there is not a selective detection among these VOCs. Figure 17 reports the response vs temperature graph of thermally oxidized ZnO nanowires for different gases. Here, ZnO_T400 and ZnO_T500 represent the ZnO nanowires grown at 400 and 500 °C, respectively.

p-type MOX semiconductors are consistently classified as secondary choices in several applications. In gas sensing, the trend is also similar.^39^ At the Sensor Laboratory, in the past few years different p-type metal oxides such as CuO^109^ and NiO^48,90,109^ have been explored. NiO exhibits better overall sensing performance comparing to CuO, even though CuO is one of the most studied p-type materials in the literature.^115−117^ More interesting, as discussed earlier, NiO nanowires were for the first time grown by vapor-phase techniques, and they were successfully integrated into gas-sensing devices.^48^ The fabricated NiO nanowire-based sensors exhibit a stronger response toward hydrogen at 300 °C; however, their response toward VOCs is very weak (Figure 18).

**Figure 19:**
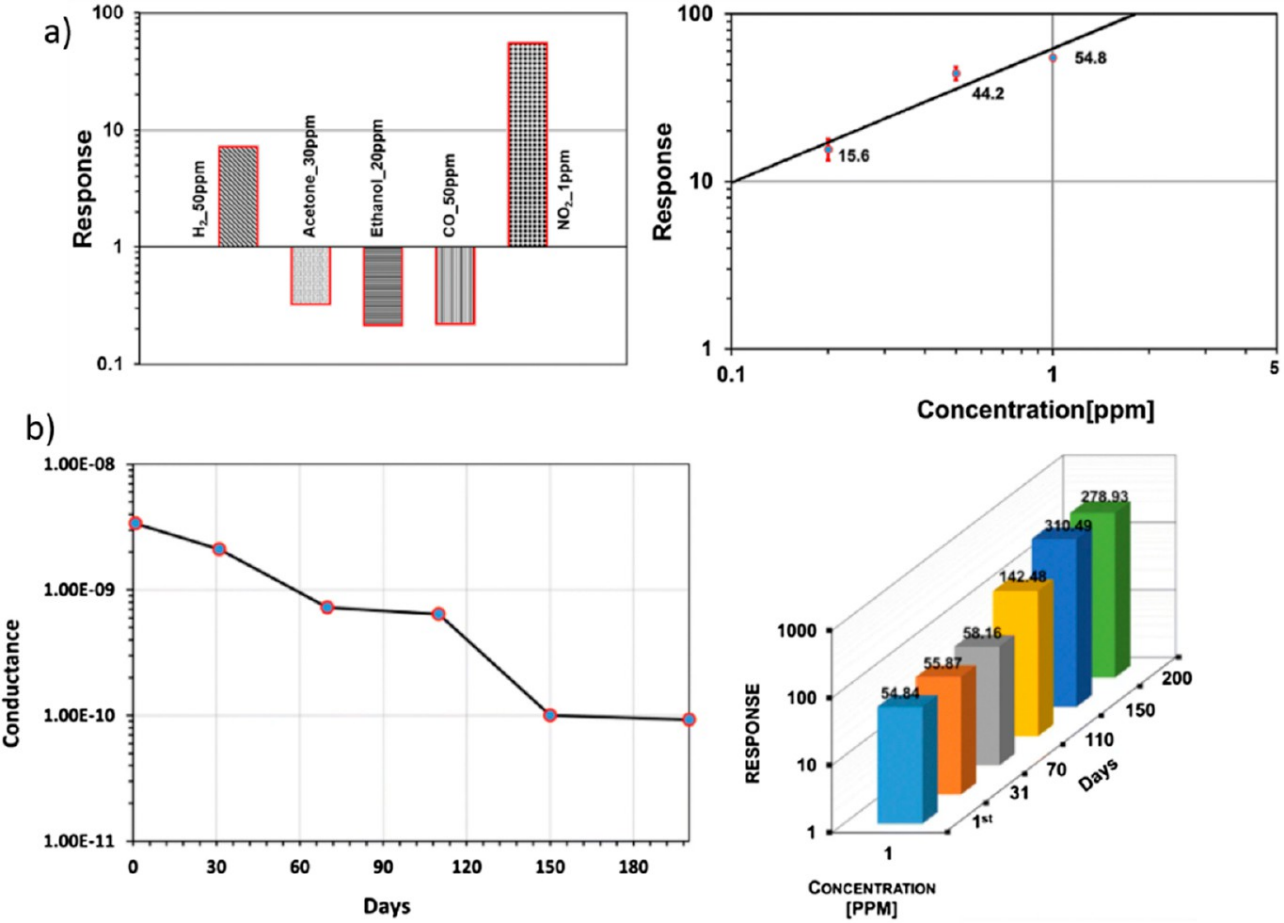
(a, Left) Cross-sensitivity response of the NiO sensor toward various gas analysts (H_2_, 50 ppm; acetone, 30 ppm; ethanol, 20 ppm; CO, 50 ppm; and NO_2_, 1 ppm). (Right) Calibration curve (response vs NO_2_ concentration) at the optimal working temperature of 200 °C. (b, Left) Trend of baseline conductance of NiO sensors on different measurement days. (Right) Sensing response of the NiO nanowire-based sensor over a period of 200 days toward 1 ppm of NO_2_. Adapted with permission from ref (78). Copyright (2019) Springer.

Furthermore, in a recent study by Kaur et al.,^78^ the detection of NO_2_ using the VLS-grown NiO nanowires is reported. These nanowires exhibited superior response and selectivity toward NO_2_ at an operating temperature of 200 °C in comparison to other gas analysts (Figure 19a). Indeed, the lower detection limit was found to be at the parts per billion level. The average exposure to NO_2_, over a period of 1 h, should be less than 0.2 ppm following the European Union (EU) Air Quality Standards.^118^ The response of NiO NWs is sufficiently higher and can easily detect such a low concentration, exhibiting a response of approximately 15 for 0.2 ppm, which makes NiO NWs a straightforward ideal candidate. Moreover, in the same article, a shelf life study of sensors stored in an ambient environment over a period of approximately 6 months (Figure 19b) is reported. The effect of atmospheric gases and humidity on the baseline conduction and their effect on the response are evidenced. The sensor performances show no significant degradation over these long-term tests.

These studies show that in the n-type MOX-dominated gas sensor world there are still chances for p-type metal oxides as a potential candidate for developing sensing devices.

In Table S2, we have compared the sensing performance of different metal oxide nanowires prepared by the Sensor Laboratory with some recent articles. For the comparison, different forms of metal oxides such as nanorods and thin films reported by different researchers have been considered to be limited data on nanowires. The sensor working temperature, type and concentration of gas analyte, sensor response, and technique used to fabricate the metal oxide nanostructures have been presented in Table S2.

## Strategies for the Improvement of the Sensing Performance of One-Dimensional Nanostructure Devices

To further enhance the sensing performances of the 1D nanostructures, new strategies such as surface functionalization NWs, developing a heterojunction with a high specific surface area, and a full depletion region are auspicious candidates.^19,80,119−122^ Many recent studies have demonstrated that sensitivity, selectivity, and other chemical sensing properties of chemoresistive metal oxide sensors can be adequately improved by engaging a secondary material to functionalize 1D nanostructures (nanowire doping,^107,108^ surface functionalization of nanowires,^80,123−128^ branch-like nanostructures,^19,129^ and core–shell nanowires^130^). For instance, as previously discussed, Nb doping in WO_3_ nanowires enhances the sensing performance with respect to H_2_.^107^ In a similar framework, surface functionalization with metal clusters and 2D materials can also be used to further improve the sensing performance. In a recent report, SnO_2_ nanowires were functionalized with a graphene oxide layer for low-temperature NO_2_ sensing in the presence of UV light.^123^

On the other hand, creating a heterojunction by combining two metal oxides on a sensing platform can bring about new possibilities and further improve the sensing performance. The fabrication of composite nanostructures, involving the integration of n-type and p-type metal oxides, allows the combination of the different individual properties into a single system. For 1D nanostructures, this can be done using various strategies and synthesis techniques, and branched heterostructures or core–shell heterostructures can easily be achieved.^131−133^

**Figure 20:**
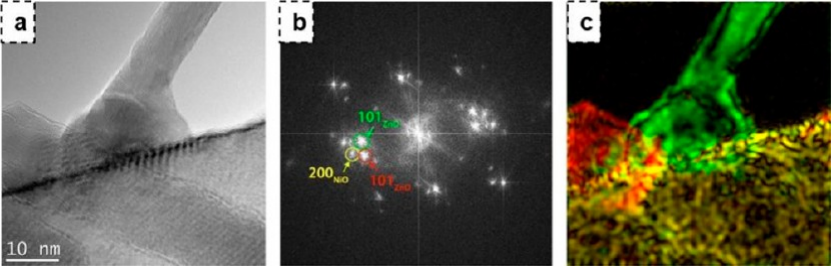
(a) HRTEM image of NiO/ZnO heterostructures. (b) FFT of heterostructure reported in (c). Colored representation of NiO and ZnO planes. Adapted with permission from ref (19). Copyright (2018) Elsevier.

Indeed, branched 1D heterostructures exhibit strong interactions between tightly packed interfaces that can improve their performances, but they are complex to predict. In 2018, Kaur et al.^19^ demonstrated the growth of NiO/ZnO branched heterostructures by a two-step vapor-phase growth method. NiO nanowires grown by VLS act as backbones for ZnO nanostructure condensation. The selected-area electron diffraction data (SAED) demonstrate that the ZnO nanowires were grown epitaxially along (101) planes on the strongly oriented NiO nanowires along (200) crystallographic planes shown in Figure 20.

**Figure 21:**
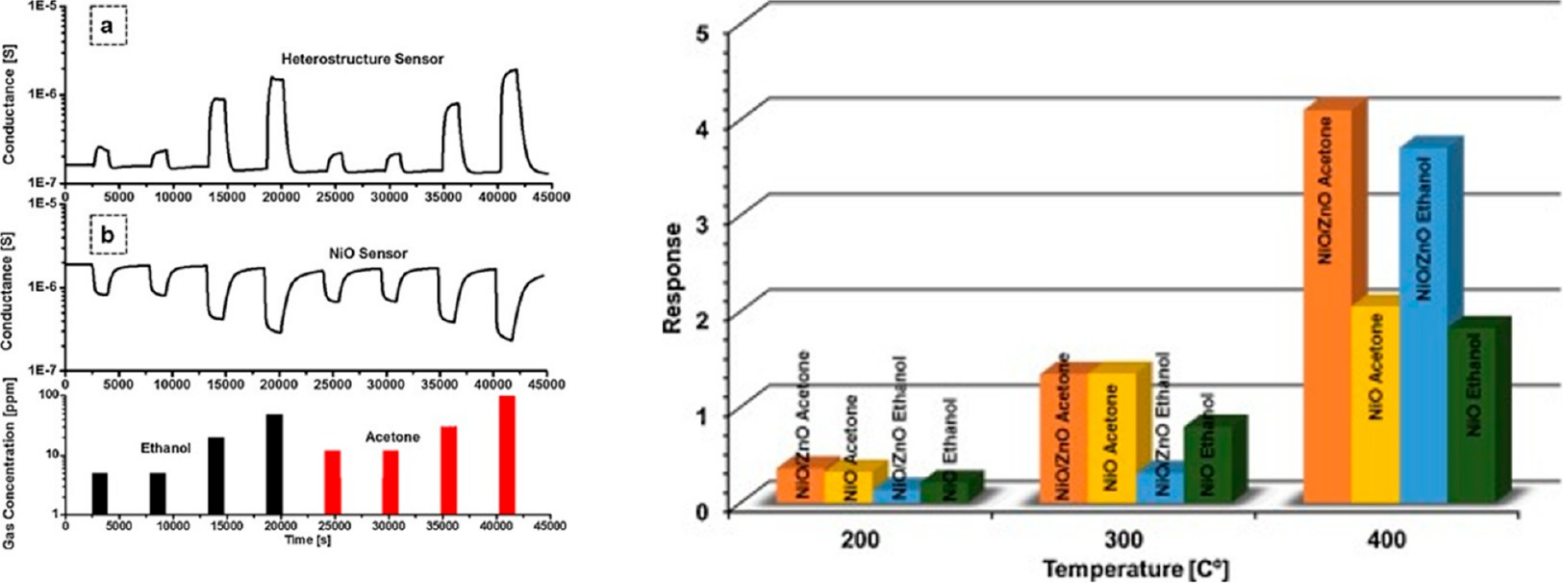
(a) Dynamic response of the NiO/ZnO heterostructure sensing device. (b, Left) Dynamic response of the NiO sensing device toward reducing gases (ethanol, black, 5–5–20–50 ppm at 400 °C; acetone, red, 10–10–30–100 ppm at 400 °C) measured at a relative humidity of 50% at 20 °C. (Right) Response of the NiO/ZnO heterostructure and NiO nanowire sensors toward target gases: NiO/ZnO acetone (brown), NiO acetone (yellow), 30 ppm; NiO/ZnO ethanol (blue), NiO ethanol (green), 20 ppm. RH 50% at 20 °C. Adapted with permission from ref (19). Copyright (2018) Elsevier.

Their sensing performances were superior to those of different VOCs such as ethanol and acetone in comparison with those of bare NiO nanowires. Calculated detection limits of 7 and 11 ppm have been found for ethanol and acetone, respectively. Furthermore, the possible interpretation given by the authors for the sensing response enhancement relies on the junction formation by charge transfer as the two Fermi levels come to equilibrium, further extending the charge depletion region. This in turn increases the overall heterostructure resistance, leading to the sensing property enhancement for the heterostructured material. The sensing performance of fabricated NiO/ZnO heterostructures compared with that of NiO nanowires is shown in Figure 21.

Furthermore, 1D-core/shell structures are another promising type of heterostructure that can be exploited in chemical sensing. Several methods were used to create core–shell morphologies.^134−138^ However, one of the most used and promising is the ALD (atomic-layer deposition) method of depositing a thin layer of a material on top of nanowires. In a recent report, the synthesis of 1D n-SnO_2_-core/p-NiO-shell nanowires (CSNWs) on an alumina substrate by using vapor phase growth (VLS) and atomic layer deposition techniques was presented.^130^

**Figure 22:**
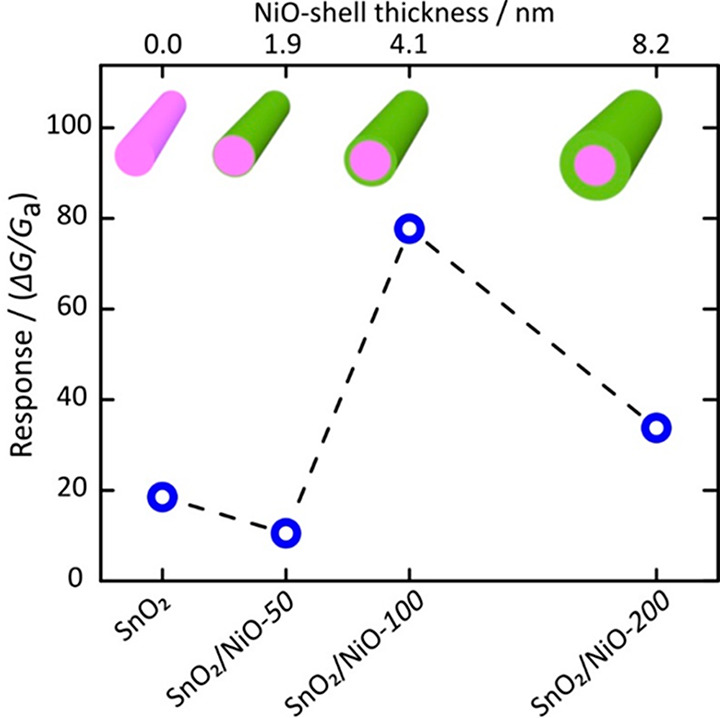
Sensing response of the SnO_2_/NiO-X CSNWs heterostructures as a function of the NiO-shell layer thickness toward 200 ppm of hydrogen at 500 °C. Adapted with permission from ref (130). Copyright (2020) American Chemical Society.

The thickness of the NiO-shell layer varies from a range of 2 to 8.2 nm. After the NiO coating on SnO_2_ NWs, the electrical conductance of the sensors decreases by many orders of magnitude. This suggested that the conductivity of the sensors is majorly dominated by Schottky barrier junctions across the interface of n–p (core/shell). The author presented the gas-sensing response of pristine SnO_2_ and SnO_2_/NiO core–shell heterostructure sensors with different NiO-shell thicknesses toward H_2_ at various temperatures. Three different ALD cycles (50, 100, and 200) were applied to modulate the thickness of NiO on SnO_2_ nanowires. Comparing the responses showed that SnO_2_/NiO-100 exhibits the best performance (shell thickness ca. 4.1 nm) due to the maximized radial modulation of the depletion region (Figure 22).

**Figure 23:**
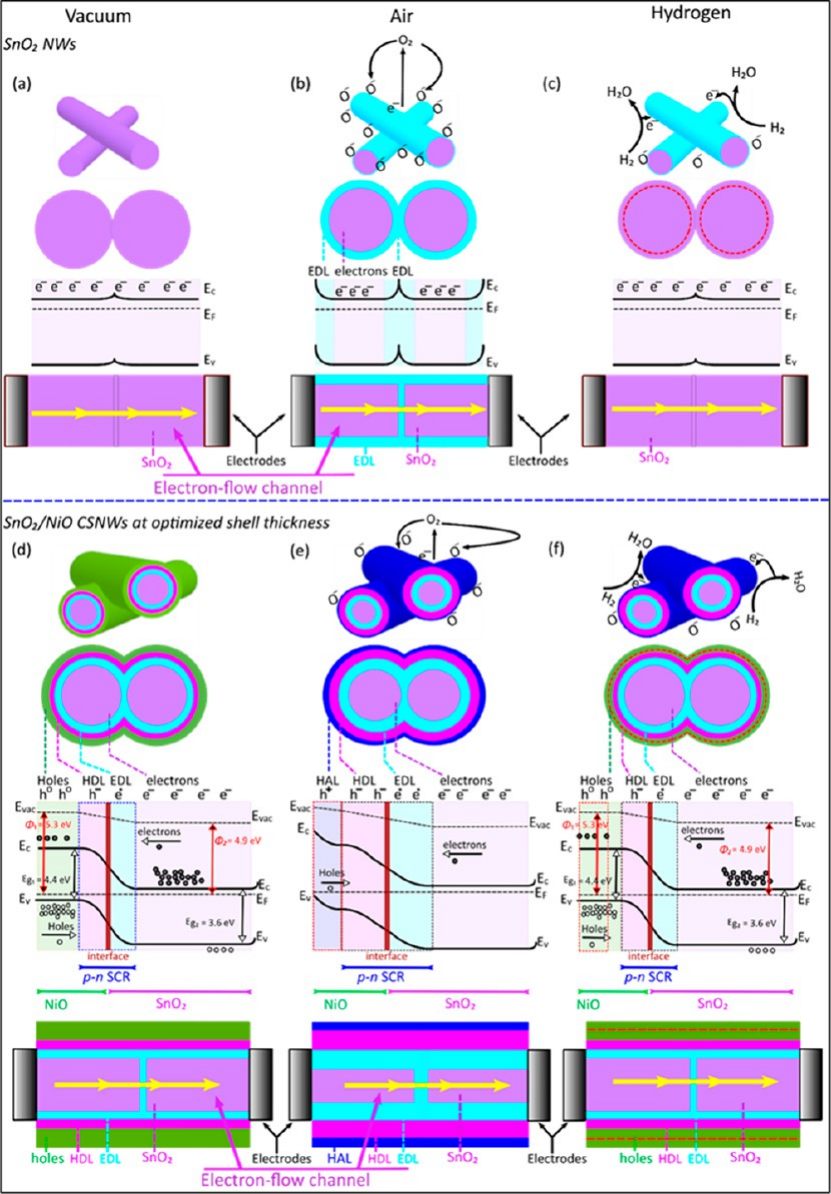
Schematics of the hydrogen-sensing mechanism, electron-flow channel, and corresponding proposed energy band diagrams for the pristine SnO_2_ NWs and SnO_2_/NiO CSNWs heterostructures. SnO_2_ NWs: (a) in vacuum, (b) in air, where the surface of the NWs is under the electron depletion effect due to the adsorption of the oxygen species, narrowing the conduction channel by creating a potential barrier height at the surfaces and the grain–grain contacts, and (c) in hydrogen, where the hydrogen reacts with the adsorbed oxygen species and releases back electrons to the SnO_2_ surface by decreasing the potential barrier height broadening the conduction channel. SnO_2_/NiO CSNWs at an optimized shell thickness: (d) in vacuum, where as the NiO shell layer is deposited onto the SnO_2_ core, a p–n junction is formed at the interface of the two materials, (e) in air, where adsorbed oxygen species withdraw electrons at the surface, creating a HAL and broadening the SCR at the p–n junction with significant narrowing of the conduction channel, and (f) in hydrogen, where the electrons released back onto the NiO surfaces decrease the width of the HAL and SCR at the p–n junction with the broadening of the conduction channel. Abbreviations used in the diagrams are the hole-accumulation layer (HAL), electron-depletion layer (EDL), hole-depletion layer (HDL), and space–charge region (SCR). Values are shown for the work functions (Φ) and energy band gaps (*E*_g_). Adapted with permission from ref (130). Copyright (2020) American Chemical Society.

The authors proposed two possible mechanisms for this behavior: (i) a hole-accumulation layer within the NiO shell and (ii) the barrier height at the interface of NiO-SnO_2_. As in the case of heterostructures, a p–n junction is created at the interface, which results in the Fermi level modulation. This induces band bending and the formation of a depletion region, which leads to an increase in resistance. As the sensor is exposed to air, an oxygen species adsorbs onto the NiO surface, trapping electrons and creating a hole-accumulation layer (HAL) in the near-surface region. The concentration of holes increased in the NiO-shell, which causes the enhancement of the charge gradient near the junction. This leads to an expansion of the depletion region and increases the barrier height at the junction. Due to this, the overall resistance of the system increases (Figure 23).

Furthermore, in the presence of H_2_, it reacts with the adsorbed oxygen ions and releases electrons back into the system. These released electrons recombine with the holes of NiO, which in turn decrease the charge carrier concentration at the junction, resulting in narrowing the space–charge region. Thus, in the presence of the NiO layer the extent in the change of conductance is higher. Another important fact to consider is the shell layer thickness because it affects the depletion region formation at the interface. The highest response was exhibited when the NiO shell thickness was 4.1 nm for the SnO_2_/NiO-100 sensors, a value similar to the hole-accumulation layer thickness, which is also related to the Debye length. Further increasing the thickness of the layer decrease the response so that the shell layer is no longer depleted. In Table S3, some interesting reports available in the literature along with our team work are presented.

## Conclusions and Future Perspectives

Metal oxide nanowire-based chemical sensors showed remarkable performance and have been explored for the detection of various analytes such as VOCs, toxic gases, environmental pollutants, and explosives. The reasons behind their astonishing performances are high crystallinity, well-defined crystal orientation, controlled electrical properties, and a high surface-to-volume ratio. In the field of 1D MOX nanostructures, the work done by our group in 2002 on the VLS synthesis of tin dioxide (SnO_2_) nanobelts for the gas-sensing application inspired many researchers, and afterward tremendous success until now has been achieved. In MOX, the chemisorption of oxygen ions creates the EDL on n-type semiconductors and HAL on p-type semiconductors, which determines their sensing behavior. By operating at different temperatures, these types of chemisorbed ions can be controlled, and the selectivity toward a particular analyte can be achieved. For example, the presence of O^–^ ions on VLS-grown WO_3_ nanowires at 200 °C enhanced their reactivity toward O_3_, while when sensors operated at 400 °C, they showed excellent performance toward H_2_S due to the dominance of O^2–^ ions on the WO_3_ surface.

The surface morphology of nanowires, which also play a major role in determining the sensing performance, is largely dependent on the growth technique used. Indeed, MOX nanowires grown using the vapor-phase mechanism were found to be highly crystalline with minimal defects and hence showed remarkable sensing performance. In MOX nanowires growth by VLS, the type of metal catalyst (generally a noble metal) affects the morphology of nanowires, which in turn influences the sensing performance. Hence, the appropriate selection of a metal catalyst in the VLS method is crucial, especially when one works on new material growth. While optimizing ZnO and NiO nanowire growth, it has been observed that the nanowires grown using Au catalysts possesses uniform and dense morphology as compared to other catalysts (Pt, Pd, etc.). On the other hand, thermal oxidation offers the possibility to grow nanowires at relatively low temperatures, which is generally not possible in vapor-phase growth.

A recent achievement of our group is the first-time growth of NiO nanowires using the VLS mechanism and integration into conductometric chemical-/gas-sensing devices for hydrogen and NO_2_ detection. Indeed, NiO nanowire chemical-/gas-sensing devices show no major degradation in their devices performance as measured for a period of 6 months. This is remarkable work in the development of NiO-based gas-sensing devices as only limited works have been presented on p-type metal oxides in comparison to n-type.

Moreover, in this feature article we presented an overview of different approaches such as the fabrication of nanowires heterostructures used to further improve the sensing performance of MOX-based gas sensors. Branched-like NiO/ZnO nanowire heterostructure sensing devices using the VLS mechanism showed superior performance compared to NiO nanowires for ethanol and acetone detection. The reason behind their superior performance was found to be the formation of junctions between NiO and ZnO, increasing the resistance and in turn enhancing the sensing performance. Moreover, the detailed microstructural investigation reveals the epitaxial growth along the (101) planes of ZnO NWs on strongly oriented NiO NWs. Furthermore, the 1D n-SnO_2_-core/p-NiO-shell nanowire heterostructure synthesized by the VLS mechanism and ALD showed excellent sensing performance toward hydrogen. The sensing mechanism reveals that the formation of the hole-accumulation layer within the NiO shell and the barrier height at the interface of NiO-SnO_2_ were the two major reasons behind their excellent sensing behavior.

However, besides the recent advance, the selectivity of the metal oxide-based gas sensor is still an issue which requires further work. We believe that with the availability of an advanced synthesis/characterization technique and other processes such as surface functionalization, this challenging issue will be partially addressed. Indeed, the surface functionalization of MOX nanostructures using self- assembled monolayers (SAM) which is majorly used in biosensing and synthesis of novel supramolecular architecture can also be adopted to improve the selectivity and performance of gas sensors. The reason behind this perspective is the nature of self-assembled monolayers such as silanes that can generate different functional groups on the oxide surface and react only to the specific gas analytes due to their functional properties. Another area that requires further investigations is the theoretical explanation of the chemical sensing mechanism, specifically for 1D nanostructured MOX as the available ones hold for granular morphology. This requires a great deal of work in the investigation of fundamental physical/chemical properties of these materials especially using operando analyses.

## Supporting Information Available

Conductometric gas-sensing measurement setup; advantages and disadvantages of vapor-phase growth and thermal oxidation growth techniques (PDF)

## References

[ref1] Ruiz C.; Garcia-Frutos E. M.; Hennrich G.; Gomez-Lor B. Organic Semiconductors toward Electronic Devices: High Mobility and Easy Processability. J. Phys. Chem. Lett. 2012, 3 (11), 1428–1436. 10.1021/jz300251u. 26285617

[ref2] Fu Y.; Zhu H.; Chen J.; Hautzinger M. P.; Zhu X.-Y.; Jin S. Metal Halide Perovskite Nanostructures for Optoelectronic Applications and the Study of Physical Properties. Nat. Rev. Mater. 2019, 4 (3), 169–188. 10.1038/s41578-019-0080-9.

[ref3] Geim A. K.; Novoselov K. S. The Rise of Graphene. Nat. Mater. 2007, 6 (3), 183–191. 10.1038/nmat1849. 17330084

[ref4] Chen R.; Lan L. Solution-Processed Metal-Oxide Thin-Film Transistors: A Review of Recent Developments. Nanotechnology 2019, 30 (31), 312001 10.1088/1361-6528/ab1860. 30974423

[ref5] Comini E. Integration of Metal Oxide Nanowires in Flexible Gas Sensing Devices. Sensors 2013, 13 (8), 10659–10673. 10.3390/s130810659. 23955436 PMC3812622

[ref6] Singh M.; Mulla M. Y.; Manoli K.; Magliulo M.; Ditaranto N.; Cioffi N.; Palazzo G.; Torsi L.; Santacroce M. V.; Di’Franco C.; Scamarcio G. Bio-Functionalization of ZnO Water Gated Thin-Film Transistors. 2015 6th International Workshop on Advances in Sensors and Interfaces (IWASI); IEEE, 2015; pp 261–265. 10.1109/IWASI.2015.7184944.

[ref7] Shin S. S.; Lee S. J.; Seok S. Il. Metal Oxide Charge Transport Layers for Efficient and Stable Perovskite Solar Cells. Adv. Funct. Mater. 2019, 29, 1900455 10.1002/adfm.201900455.

[ref8] Dargar S. K.; Srivastava V. M. Design and Analysis of IGZO Thin Film Transistor for AMOLED Pixel Circuit Using Double-Gate Tri Active Layer Channel. Heliyon 2019, 5 (4), e01452 10.1016/j.heliyon.2019.e01452. 31008392 PMC6458471

[ref9] Fortunato E.; Barquinha P.; Martins R. Oxide Semiconductor Thin-Film Transistors: A Review of Recent Advances. Adv. Mater. 2012, 24, 2945–2986. 10.1002/adma.201103228. 22573414

[ref10] Hosono H. Transparent Oxide Semiconductors: Fundamentals and Recent Progress. Transparent Electronics; John Wiley & Sons: 2010; pp 31–59 10.1002/9780470710609.ch2.

[ref11] Fortunato E.; Hosono H.; Granqvist C.; Wager J. Advances in Transparent Electronics: From Materials to Devices I. Thin Solid Films 2008, 516 (7), 1313 10.1016/j.tsf.2007.09.040.

[ref12] Coll M.; Fontcuberta J.; Althammer M.; Bibes M.; Boschker H.; Calleja A.; Cheng G.; Cuoco M.; Dittmann R.; Dkhil B.; El Baggari I.; Fanciulli M.; Fina I.; Fortunato E.; Frontera C.; Fujita S.; Garcia V.; Goennenwein S. T. B.; Granqvist C.-G.; Grollier J.; Gross R.; Hagfeldt A.; Herranz G.; Hono K.; Houwman E.; Huijben M.; Kalaboukhov A.; Keeble D. J.; Koster G.; Kourkoutis L. F.; Levy J.; Lira-Cantu M.; MacManus-Driscoll J. L.; Mannhart J.; Martins R.; Menzel S.; Mikolajick T.; Napari M.; Nguyen M. D.; Niklasson G.; Paillard C.; Panigrahi S.; Rijnders G.; Sánchez F.; Sanchis P.; Sanna S.; Schlom D. G.; Schroeder U.; Shen K. M.; Siemon A.; Spreitzer M.; Sukegawa H.; Tamayo R.; van den Brink J.; Pryds N.; Granozio F. M. Towards Oxide Electronics: A Roadmap. Appl. Surf. Sci. 2019, 482, 1–93. 10.1016/j.apsusc.2019.03.312.

[ref13] Yu X.; Marks T. J.; Facchetti A. Metal Oxides for Optoelectronic Applications. Nat. Mater. 2016, 15, 383 10.1038/nmat4599. 27005918

[ref14] Maduraiveeran G.; Sasidharan M.; Jin W. Earth-Abundant Transition Metal and Metal Oxide Nanomaterials: Synthesis and Electrochemical Applications. Prog. Mater. Sci. 2019, 106, 100574 10.1016/j.pmatsci.2019.100574.

[ref15] Široký K.; Jirešová J. A Novel SnO2-Based Gas Sensor. Talanta 1994, 41 (10), 1735–1740. 10.1016/0039-9140(94)E0118-B. 18966127

[ref16] B rsan N.; Weimar U. Understanding the Fundamental Principles of Metal Oxide Based Gas Sensors; the Example of CO Sensing with SnO2sensors in the Presence of Humidity. J. Phys.: Condens. Matter 2003, 15 (20), R813–R839. 10.1088/0953-8984/15/20/201.

[ref17] Koziej D.; Hübner M.; Barsan N.; Weimar U.; Sikora M.; Grunwaldt J.-D. Operando X-Ray Absorption Spectroscopy Studies on Pd-SnO2 Based Sensors. Phys. Chem. Chem. Phys. 2009, 11 (38), 8620–8625. 10.1039/b906829e. 19774296

[ref18] Gründler P. Chemical Sensors. ChemTexts 2017, 3 (4), 16 10.1007/s40828-017-0052-x.

[ref19] Kaur N.; Zappa D.; Ferroni M.; Poli N.; Campanini M.; Negrea R.; Comini E. Branch-like NiO/ZnO Heterostructures for VOC Sensing. Sens. Actuators, B 2018, 262, 477–485. 10.1016/j.snb.2018.02.042.

[ref20] Oberegger S. P.; Jones O. A. H.; Spencer M. J. S. Effect of Nanostructuring of ZnO for Gas Sensing of Nitrogen Dioxide. Comput. Mater. Sci. 2017, 132, 104–115. 10.1016/j.commatsci.2017.02.019.

[ref21] Kotsikau D.; Ivanovskaya M. In Metal Oxide Semiconductor Sensors for Detection of Toxic and Explosive Gases BT - Electronic Noses & Sensors for the Detection of Explosives; Gardner J. W., Yinon J., Eds.; Springer: Dordrecht, The Netherlands, 2004; pp 93–115.

[ref22] Kanan S. M.; El-Kadri O. M.; Abu-Yousef I. A.; Kanan M. C. Semiconducting Metal Oxide Based Sensors for Selective Gas Pollutant Detection. Sensors 2009, 9, 8158–8196. 10.3390/s91008158. 22408500 PMC3292102

[ref23] Comini E.; Sberveglieri G. Metal Oxide Nanowires as Chemical Sensors. Mater. Today 2010, 13 (7–8), 36–44. 10.1016/S1369-7021(10)70126-7.

[ref24] Nunes D.; Pimentel A.; Gonçalves A.; Pereira S.; Branquinho R.; Barquinha P.; Fortunato E.; Martins R. Metal Oxide Nanostructures for Sensor Applications. Semicond. Sci. Technol. 2019, 34 (4), 043001 10.1088/1361-6641/ab011e.

[ref25] Kim D. H.; Shim Y.-S.; Jeon J.-M.; Jeong H. Y.; Park S. S.; Kim Y.-W.; Kim J.-S.; Lee J.-H.; Jang H. W. Vertically Ordered Hematite Nanotube Array as an Ultrasensitive and Rapid Response Acetone Sensor. ACS Appl. Mater. Interfaces 2014, 6 (17), 14779–14784. 10.1021/am504156w. 25157784

[ref26] Shingange K.; Tshabalala Z. P.; Ntwaeaborwa O. M.; Motaung D. E.; Mhlongo G. H. Highly Selective NH3 Gas Sensor Based on Au Loaded ZnO Nanostructures Prepared Using Microwave-Assisted Method. J. Colloid Interface Sci. 2016, 479, 127–138. 10.1016/j.jcis.2016.06.046. 27388126

[ref27] Hoffmann M. W. G.; Mayrhofer L.; Casals O.; Caccamo L.; Hernandez-Ramirez F.; Lilienkamp G.; Daum W.; Moseler M.; Waag A.; Shen H.; Prades J. D. A Highly Selective and Self-Powered Gas Sensor Via Organic Surface Functionalization of p-Si/n-ZnO Diodes. Adv. Mater. 2014, 26 (47), 8017–8022. 10.1002/adma.201403073. 25355241

[ref28] Comini E.; Faglia G.; Sberveglieri G.; Pan Z.; Wang Z. L. Stable and Highly Sensitive Gas Sensors Based on Semiconducting Oxide Nanobelts. Appl. Phys. Lett. 2002, 81 (10), 1869–1871. 10.1063/1.1504867.

[ref29] Calavia R.; Mozalev A.; Vazquez R.; Gracia I.; Cané C.; Ionescu R.; Llobet E. Fabrication of WO3 Nanodot-Based Microsensors Highly Sensitive to Hydrogen. Sens. Actuators, B 2010, 149 (2), 352–361. 10.1016/j.snb.2010.06.055.

[ref30] Zappa D.; Bertuna A.; Comini E.; Herold M.; Poli N.; Sberveglieri G. Tungsten Oxide Nanowires on Micro Hotplates for Gas Sensing Applications. Procedia Eng. 2015, 120, 439–442. 10.1016/j.proeng.2015.08.663.

[ref31] Zhao Y.; Li C.; Chen M.; Yu X.; Chang Y.; Chen A.; Zhu H.; Tang Z. Growth of Aligned ZnO Nanowires via Modified Atmospheric Pressure Chemical Vapor Deposition. Phys. Lett. A 2016, 380 (47), 3993–3997. 10.1016/j.physleta.2016.06.030.

[ref32] Thiemann S.; Gruber M.; Lokteva I.; Hirschmann J.; Halik M.; Zaumseil J. High-Mobility ZnO Nanorod Field-Effect Transistors by Self-Alignment and Electrolyte-Gating. ACS Appl. Mater. Interfaces 2013, 5 (5), 1656–1662. 10.1021/am3026739. 23398625

[ref33] Arafat M. M.; Dinan B.; Akbar S. A.; Haseeb A. S. M. A. Gas Sensors Based on One Dimensional Nanostructured Metal-Oxides: A Review. Sensors 2012, 12 (6), 7207–7258. 10.3390/s120607207. 22969344 PMC3435973

[ref34] Comini E. Metal Oxide Nanowire Chemical Sensors: Innovation and Quality of Life. Mater. Today 2016, 19 (10), 559–567. 10.1016/j.mattod.2016.05.016.

[ref35] Karnati P.; Akbar S.; Morris P. A. Conduction Mechanisms in One Dimensional Core-Shell Nanostructures for Gas Sensing: A Review. Sens. Actuators, B 2019, 295, 127–143. 10.1016/j.snb.2019.05.049.

[ref36] Miller D. R.; Akbar S. A.; Morris P. A. Nanoscale Metal Oxide-Based Heterojunctions for Gas Sensing: A Review. Sens. Actuators, B 2014, 204, 250–272. 10.1016/j.snb.2014.07.074.

[ref37] Walker J. M.; Akbar S. A.; Morris P. A. Synergistic Effects in Gas Sensing Semiconducting Oxide Nano-Heterostructures: A Review. Sens. Actuators, B 2019, 286, 624–640. 10.1016/j.snb.2019.01.049.

[ref38] Spear W. E.; Le Comber P. G. Substitutional Doping of Amorphous Silicon. Solid State Commun. 1993, 88 (11–12), 1015–1018. 10.1016/0038-1098(93)90286-V.

[ref39] Kim H.-J.; Lee J.-H. Highly Sensitive and Selective Gas Sensors Using P-Type Oxide Semiconductors: Overview. Sens. Actuators, B 2014, 192, 607–627. 10.1016/j.snb.2013.11.005.

[ref40] Barsan N.; Weimar U. Conduction Model of Metal Oxide Gas Sensors. J. Electroceram. 2001, 7 (3), 143–167. 10.1023/A:1014405811371.

[ref41] Comini E.; Baratto C.; Faglia G.; Ferroni M.; Vomiero A.; Sberveglieri G. Quasi-One Dimensional Metal Oxide Semiconductors: Preparation, Characterization and Application as Chemical Sensors. Prog. Mater. Sci. 2009, 54 (1), 1–67. 10.1016/j.pmatsci.2008.06.003.

[ref42] Li Z.; Li H.; Wu Z.; Wang M.; Luo J.; Torun H.; Hu P.; Yang C.; Grundmann M.; Liu X.; Fu Y. Advances in Designs and Mechanisms of Semiconducting Metal Oxide Nanostructures for High-Precision Gas Sensors Operated at Room Temperature. Mater. Horiz. 2019, 6 (3), 470–506. 10.1039/C8MH01365A.

[ref43] Wang C.; Yin L.; Zhang L.; Xiang D.; Gao R. Metal Oxide Gas Sensors: Sensitivity and Influencing Factors. Sensors 2010, 10 (3), 2088–2106. 10.3390/s100302088. 22294916 PMC3264469

[ref44] Iwamoto M.; Yoda Y.; Yamazoe N.; Seiyama T. Study of Metal Oxide Catalysts by Temperature Programmed Desorption. 4. Oxygen Adsorption on Various Metal Oxides. J. Phys. Chem. 1978, 82 (24), 2564–2570. 10.1021/j100513a006.

[ref45] Comini E. Metal Oxide Nano-Crystals for Gas Sensing. Anal. Chim. Acta 2006, 568 (1), 28–40. 10.1016/j.aca.2005.10.069. 17761243

[ref46] Barsan N.; Simion C.; Heine T.; Pokhrel S.; Weimar U. Modeling of Sensing and Transduction for P-Type Semiconducting Metal Oxide Based Gas Sensors. J. Electroceram. 2010, 25 (1), 11–19. 10.1007/s10832-009-9583-x.

[ref47] Hübner M.; Simion C. E.; Tomescu-Stanoiu A.; Pokhrel S.; Bârsan N.; Weimar U. Influence of Humidity on CO Sensing with P-Type CuO Thick Film Gas Sensors. Sens. Actuators, B 2011, 153 (2), 347–353. 10.1016/j.snb.2010.10.046.

[ref48] Kaur N.; Comini E.; Zappa D.; Poli N.; Sberveglieri G. Nickel Oxide Nanowires: Vapor Liquid Solid Synthesis and Integration into a Gas Sensing Device. Nanotechnology 2016, 27 (20), 205701 10.1088/0957-4484/27/20/205701. 27053627

[ref49] Can I.; Weimar U.; Barsan N. Operando Investigations of Differently Prepared In2O3-Gas Sensors. Proceedings 2017, 1 (4), 432 10.3390/proceedings1040432.

[ref50] Pokhrel S.; Simion C. E.; Quemener V.; Bârsan N.; Weimar U. Investigations of Conduction Mechanism in Cr2O3 Gas Sensing Thick Films by Ac Impedance Spectroscopy and Work Function Changes Measurements. Sens. Actuators, B 2008, 133 (1), 78–83. 10.1016/j.snb.2008.01.054.

[ref51] Korotcenkov G.; Brinzari V.; Cho B. K. Conductometric Gas Sensors Based on Metal Oxides Modified with Gold Nanoparticles: A Review. Microchim. Acta 2016, 183 (3), 1033–1054. 10.1007/s00604-015-1741-z.

[ref52] Sberveglleri G. In Gas Sensors: Principles, Operation and Developments; Sberveglieri G., Ed.; Springer: The Netherlands, 1992. 10.1088/0957-0233/6/7/021.

[ref53] Korotcenkov G. In Handbook of Gas Sensor Materials; Korotcenkov G., Ed.; Springer-Verlag: New York, 2014. 10.1007/978-1-4614-7388-6.

[ref54] Korotcenkov G. Metal Oxides for Solid-State Gas Sensors: What Determines Our Choice?. Mater. Sci. Eng., B 2007, 139 (1), 1–23. 10.1016/j.mseb.2007.01.044.

[ref55] Aswal D. K.; Gupta S. K. In Science and Technology of Chemiresistor Gas Sensors; Gupta S. K., Ed.; Nova Publishers: 2007.

[ref56] Mijatovic D.; Eijkel J. C. T.; van den Berg A. Technologies for Nanofluidic Systems: Top-down vs. Bottom-up—a Review. Lab Chip 2005, 5 (5), 492–500. 10.1039/b416951d. 15856084

[ref57] Candeloro P.; Comini E.; Baratto C.; Faglia G.; Sberveglieri G.; Kumar R.; Carpentiero A.; Fabrizio E. Di. SnO[Sub 2] Lithographic Processing for Nanopatterned Gas Sensors. J. Vac. Sci. Technol., B: Microelectron. Process. Phenom. 2005, 23 (6), 2784 10.1116/1.2110371.

[ref58] Corbierre M. K.; Beerens J.; Lennox R. B. Gold Nanoparticles Generated by Electron Beam Lithography of Gold(I)-Thiolate Thin Films. Chem. Mater. 2005, 17, 5774 10.1021/cm051085b.

[ref59] Chmielewski A. G.; Chmielewska D. K.; Michalik J.; Sampa M. H. Prospects and Challenges in Application of Gamma, Electron and Ion Beams in Processing of Nanomaterials. Nucl. Instrum. Methods Phys. Res., Sect. B 2007, 265 (1), 339–346. 10.1016/j.nimb.2007.08.069.

[ref60] Ra H.-W.; Choi K.-S.; Kim J.-H.; Hahn Y.-B.; Im Y.-H. Fabrication of ZnO Nanowires Using Nanoscale Spacer Lithography for Gas Sensors. Small 2008, 4 (8), 1105–1109. 10.1002/smll.200700922. 18615418

[ref61] M. Nuzaihan M. N.; Hashim U.; Md Arshad M. K.; Ruslinda A. R.; Rahman S. F. A.; Fathil M. F. M.; Ismail M. H. Top-Down Nanofabrication and Characterization of 20 Nm Silicon Nanowires for Biosensing Applications. PLoS One 2016, 11 (3), e0152318 10.1371/journal.pone.0152318. 27022732 PMC4811568

[ref62] Park I.; Li Z.; Pisano A. P.; Williams R. S. Top-down Fabricated Silicon Nanowire Sensors for Real-Time Chemical Detection. Nanotechnology 2010, 21 (1), 015501 10.1088/0957-4484/21/1/015501. 19946164

[ref63] Tian W.-C.; Ho Y.-H.; Chen C.-H.; Kuo C.-Y. Sensing Performance of Precisely Ordered TiO2 Nanowire Gas Sensors Fabricated by Electron-Beam Lithography. Sensors 2013, 13 (1), 865–874. 10.3390/s130100865. 23344381 PMC3574709

[ref64] Kim H. W.; Na H. G.; Kwon Y. J.; Cho H. Y.; Lee C. Decoration of Co Nanoparticles on ZnO-Branched SnO2 Nanowires to Enhance Gas Sensing. Sens. Actuators, B 2015, 219, 22–29. 10.1016/j.snb.2015.05.017.

[ref65] Duoc V. T.; Le D. T. T.; Hoa N. D.; Van Duy N.; Hung C. M.; Nguyen H.; Van Hieu N. New Design of ZnO Nanorod- and Nanowire-Based NO_2_ Room-Temperature Sensors Prepared by Hydrothermal Method. J. Nanomater. 2019, 2019, 6821937 10.1155/2019/6821937.

[ref66] Wu G. S.; Xie T.; Yuan X. Y.; Li Y.; Yang L.; Xiao Y. H.; Zhang L. D. Controlled Synthesis of ZnO Nanowires or Nanotubes via Sol-Gel Template Process. Solid State Commun. 2005, 134 (7), 485–489. 10.1016/j.ssc.2005.02.015.

[ref67] Wu W.-Y.; Ting J.-M.; Huang P.-J. Electrospun ZnO Nanowires as Gas Sensors for Ethanol Detection. Nanoscale Res. Lett. 2009, 4 (6), 513 10.1007/s11671-009-9271-4. 20596477 PMC2893837

[ref68] Wagner R. S.; Ellis W. C. Vapor-Liquid-Solid Mechanism of Single Crystal Growth. Appl. Phys. Lett. 1964, 4 (5), 89–90. 10.1063/1.1753975.

[ref69] Hsu Y.-J.; Lu S.-Y. Vapor-Solid Growth of Sn Nanowires: Growth Mechanism and Superconductivity. J. Phys. Chem. B 2005, 109 (10), 4398–4403. 10.1021/jp046354k. 16851508

[ref70] Vomiero A.; Bianchi S.; Comini E.; Faglia G.; Ferroni M.; Sberveglieri G. Controlled Growth and Sensing Properties of In _2_ O _3_ Nanowires. Cryst. Growth Des. 2007, 7 (12), 2500–2504. 10.1021/cg070209p.

[ref71] Givargizov E. I. Fundamental Aspects of VLS Growth. J. Cryst. Growth 1975, 31, 20–30. 10.1016/0022-0248(75)90105-0.

[ref72] Zappa D.; Bertuna A.; Comini E.; Kaur N.; Poli N.; Sberveglieri V.; Sberveglieri G. Metal Oxide Nanostructures: Preparation, Characterization and Functional Applications as Chemical Sensors. Beilstein J. Nanotechnol. 2017, 8 (1), 1205–1217. 10.3762/bjnano.8.122. 28685121 PMC5480349

[ref73] Suzuki M.; Hidaka Y.; Yanagida T.; Klamchuen A.; Kanai M.; Kawai T.; Kai S. Essential Role of Catalyst in Vapor-Liquid-Solid Growth of Compounds. Phys. Rev. E 2011, 83 (6), 61606 10.1103/PhysRevE.83.061606. 21797379

[ref74] Kolasinski K. W. Catalytic Growth of Nanowires: Vapor-Liquid-Solid, Vapor-Solid-Solid, Solution-Liquid-Solid and Solid-Liquid-Solid Growth. Curr. Opin. Solid State Mater. Sci. 2006, 10 (3), 182–191. 10.1016/j.cossms.2007.03.002.

[ref75] Choi H.-J. Vapor-Liquid-Solid Growth of Semiconductor Nanowires; Springer: Berlin, 2012; pp 1–36 10.1007/978-3-642-22480-5_1.

[ref76] Roper S. M.; Davis S. H.; Norris S. A.; Golovin A. A.; Voorhees P. W.; Weiss M. Steady Growth of Nanowires via the Vapor-Liquid-Solid Method. J. Appl. Phys. 2007, 102 (3), 034304 10.1063/1.2761836.

[ref77] Kaur N.; Zappa D.; Poli N.; Comini E. Integration of VLS-Grown WO _3_ Nanowires into Sensing Devices for the Detection of H _2_ S and O _3_. ACS Omega 2019, 4 (15), 16336–16343. 10.1021/acsomega.9b01792. 31616811 PMC6787887

[ref78] Kaur N.; Zappa D.; Comini E. Shelf Life Study of NiO Nanowire Sensors for NO2 Detection. Electron. Mater. Lett. 2019, 15 (6), 743–749. 10.1007/s13391-019-00172-5.

[ref79] Comini E.; Faglia G.; Ferroni M.; Sberveglieri G. Gas Sensing Properties of Zinc Oxide Nanostructures Prepared by Thermal Evaporation. Appl. Phys. A: Mater. Sci. Process. 2007, 88 (1), 45–48. 10.1007/s00339-007-3978-9.

[ref80] Waclawik E. R.; Chang J.; Ponzoni A.; Concina I.; Zappa D.; Comini E.; Motta N.; Faglia G.; Sberveglieri G. Functionalised Zinc Oxide Nanowire Gas Sensors: Enhanced NO _2_ Gas Sensor Response by Chemical Modification of Nanowire Surfaces. Beilstein J. Nanotechnol. 2012, 3 (1), 368–377. 10.3762/bjnano.3.43. 23016141 PMC3388361

[ref81] Baratto C.; Todros S.; Faglia G.; Comini E.; Sberveglieri G.; Lettieri S.; Santamaria L.; Maddalena P. Luminescence Response of ZnO Nanowires to Gas Adsorption. Sens. Actuators, B 2009, 140 (2), 461–466. 10.1016/j.snb.2009.05.018.

[ref82] Comini E.; Baratto C.; Faglia G.; Ferroni M.; Sberveglieri G. Single Crystal ZnO Nanowires as Optical and Conductometric Chemical Sensor. J. Phys. D: Appl. Phys. 2007, 40 (23), 7255–7259. 10.1088/0022-3727/40/23/S08.

[ref83] Sberveglieri G.; Baratto C.; Comini E.; Faglia G.; Ferroni M.; Pardo M.; Ponzoni A.; Vomiero A. Semiconducting Tin Oxide Nanowires and Thin Films for Chemical Warfare Agents Detection. Thin Solid Films 2009, 517 (22), 6156–6160. 10.1016/j.tsf.2009.04.004.

[ref84] Arbiol J.; Comini E.; Faglia G.; Sberveglieri G.; Morante J. R. Orthorhombic Pbcn SnO2 Nanowires for Gas Sensing Applications. J. Cryst. Growth 2008, 310 (1), 253–260. 10.1016/j.jcrysgro.2007.10.024.

[ref85] Comini E.; Bianchi S.; Faglia G.; Ferroni M.; Vomiero A.; Sberveglieri G. Functional Nanowires of Tin Oxide. Appl. Phys. A: Mater. Sci. Process. 2007, 89 (1), 73–76. 10.1007/s00339-007-4042-5.

[ref86] Sitarz M.; Kwoka M.; Comini E.; Zappa D.; Szuber J. Surface Chemistry of SnO2 Nanowires on Ag-Catalyst-Covered Si Substrate Studied Using XPS and TDS Methods. Nanoscale Res. Lett. 2014, 9 (1), 43 10.1186/1556-276X-9-43. 24461127 PMC3913376

[ref87] Bianchi S.; Comini E.; Ferroni M.; Faglia G.; Vomiero A.; Sberveglieri G. Indium Oxide Quasi-Monodimensional Low Temperature Gas Sensor. Sens. Actuators, B 2006, 118 (1–2), 204–207. 10.1016/j.snb.2006.04.023.

[ref88] Vomiero A.; Bianchi S.; Comini E.; Faglia G.; Ferroni M.; Poli N.; Sberveglieri G. In2O3 Nanowires for Gas Sensors: Morphology and Sensing Characterisation. Thin Solid Films 2007, 515 (23), 8356–8359. 10.1016/j.tsf.2007.03.034.

[ref89] Vomiero A.; Ferroni M.; Comini E.; Faglia G.; Sberveglieri G. Insight into the Formation Mechanism of One-Dimensional Indium Oxide Wires. Cryst. Growth Des. 2010, 10 (1), 140–145. 10.1021/cg900749j.

[ref90] Kaur N.; Comini E.; Poli N.; Zappa D.; Sberveglieri G. Nickel Oxide Nanowires Growth by VLS Technique for Gas Sensing Application. Procedia Eng. 2015, 120, 760–763. 10.1016/j.proeng.2015.08.805.

[ref91] Lee S. W.; Tsai P. P.; Chen H. Comparison Study of SnO2 Thin- and Thick-Film Gas Sensors. Sens. Actuators, B 2000, 67 (1–2), 122–127. 10.1016/S0925-4005(00)00390-7.

[ref92] Di Giulio M.; Micocci G.; Serra A.; Tepore A.; Rella R.; Siciliano P. SnO2 Thin Films for Gas Sensor Prepared by r.f. Reactive Sputtering. Sens. Actuators, B 1995, 25 (1–3), 465–468. 10.1016/0925-4005(94)01397-7.

[ref93] Kwoka M.; Lyson-Sypien B.; Kulis A.; Zappa D.; Comini E. Surface Properties of SnO2 Nanowires Deposited on Si Substrate Covered by Au Catalyst Studies by XPS, TDS and SEM. Nanomaterials 2018, 8 (9), 738 10.3390/nano8090738. PMC6164749 30231566

[ref94] Karakuscu A.; Ponzoni A.; Comini E.; Sberveglieri G.; Vakifahmetoglu C. SiC Foams Decorated with SnO _2_ Nanostructures for Room Temperature Gas Sensing. Int. J. Appl. Ceram. Technol. 2014, 11 (5), 851–857. 10.1111/ijac.12295.

[ref95] Zappa D.; Melloni R.; Maraloiu V.-A.; Poli N.; Rizzoni M.; Sberveglieri V.; Sisman O.; Soprani M.; Comini E. Influence of Metal Catalyst on SnO2 Nanowires Growth and Gas Sensing Performance. Proceedings 2017, 1 (4), 460 10.3390/proceedings1040460.

[ref96] Lettieri S.; Santamaria Amato L.; Maddalena P.; Comini E.; Baratto C.; Todros S. Recombination Dynamics of Deep Defect States in Zinc Oxide Nanowires. Nanotechnology 2009, 20 (17), 175706 10.1088/0957-4484/20/17/175706. 19420601

[ref97] Jacopin G.; Rigutti L.; Bugallo A. D. L.; Julien F. H.; Baratto C.; Comini E.; Ferroni M.; Tchernycheva M. High Degree of Polarization of the Near-Band-Edge Photoluminescence in ZnO Nanowires. Nanoscale Res. Lett. 2011, 6 (1), 501 10.1186/1556-276X-6-501. 21854578 PMC3212016

[ref98] Baratto C.; Comini E.; Ferroni M.; Faglia G.; Sberveglieri G. Plasma-Induced Enhancement of UV Photoluminescence in ZnO Nanowires. CrystEngComm 2013, 15 (39), 7981 10.1039/c3ce41055b.

[ref99] Hadia N. M. A.; Alqahtani M. S.; Mohamed S. H. WO3 Nanowires for Optoelectronic and Gas Sensing Applications. Appl. Phys. A: Mater. Sci. Process. 2015, 119 (4), 1261–1267. 10.1007/s00339-015-9090-7.

[ref100] Arafat M. M.; Haseeb A. S. M. A.; Dinan B.; Akbar S. A. Stress Enhanced TiO2 Nanowire Growth on Ti-6Al-4V Particles by Thermal Oxidation. Ceram. Int. 2013, 39 (6), 6517–6526. 10.1016/j.ceramint.2013.01.084.

[ref101] Dinan B. J.; Dregia S. A.; Akbar S. A. Growth of Coaxial Nanowires by Thermal Oxidation of Ti64 Alloy. Mater. Technol. 2013, 28 (5), 280–285. 10.1179/1753555713Y.0000000059.

[ref102] Dinan B. E. N.; Akbar S. A. One-Dimensional Oxide Nanostructures Produced by Gas Phase Reaction. Funct. Mater. Lett. 2009, 02 (03), 87–94. 10.1142/S1793604709000624.

[ref103] Gonçalves A. M. B.; Campos L. C.; Ferlauto A. S.; Lacerda R. G. On the Growth and Electrical Characterization of CuO Nanowires by Thermal Oxidation. J. Appl. Phys. 2009, 106 (3), 034303 10.1063/1.3187833.

[ref104] Xu C. H.; Woo C. H.; Shi S. Q. Formation of CuO Nanowires on Cu Foil. Chem. Phys. Lett. 2004, 399 (1–3), 62–66. 10.1016/j.cplett.2004.10.005.

[ref105] Zappa D.; Comini E.; Sberveglieri G. Thermally Oxidized Zinc Oxide Nanowires for Use as Chemical Sensors. Nanotechnology 2013, 24 (44), 444008 10.1088/0957-4484/24/44/444008. 24113169

[ref106] Zappa D.; Bertuna A.; Comini E.; Molinari M.; Poli N.; Sberveglieri G. Tungsten Oxide Nanowires for Chemical Detection. Anal. Methods 2015, 7 (5), 2203–2209. 10.1039/C4AY02637C.

[ref107] Zappa D. The Influence of Nb on the Synthesis of WO3 Nanowires and the Effects on Hydrogen Sensing Performance. Sensors 2019, 19 (10), 2332 10.3390/s19102332. PMC6567310 31137592

[ref108] Zappa D.; Bertuna A.; Comini E.; Poli N.; Sberveglieri G. Influence of Nb-Doping on Hydrogen Sensing Performance of WO3 Nanowires. Procedia Eng. 2016, 168, 317–320. 10.1016/j.proeng.2016.11.205.

[ref109] Zappa D.; Comini E.; Zamani R.; Arbiol J.; Morante J. R.; Sberveglieri G. Preparation of Copper Oxide Nanowire-Based Conductometric Chemical Sensors. Sens. Actuators, B 2013, 182, 7–15. 10.1016/j.snb.2013.02.076.

[ref110] Singh M.; Manoli K.; Tiwari A.; Ligonzo T.; Di Franco C.; Cioffi N.; Palazzo G.; Scamarcio G.; Torsi L. The Double Layer Capacitance of Ionic Liquids for Electrolyte Gating of ZnO Thin Film Transistors and Effect of Gate Electrodes. J. Mater. Chem. C 2017, 5 (14), 3509–3518. 10.1039/C7TC00800G.

[ref111] Ali G. M.; Chakrabarti P. ZnO-Based Interdigitated MSM and MISIM Ultraviolet Photodetectors. J. Phys. D: Appl. Phys. 2010, 43 (41), 415103 10.1088/0022-3727/43/41/415103.

[ref112] Zappa D.; Comini E.; Zamani R.; Arbiol J.; Morante J. R.; Sberveglieri G. Copper Oxide Nanowires Prepared by Thermal Oxidation for Chemical Sensing. Procedia Eng. 2011, 25, 753–756. 10.1016/j.proeng.2011.12.185.

[ref113] Jiang X.; Herricks T.; Xia Y. CuO Nanowires Can Be Synthesized by Heating Copper Substrates in Air. Nano Lett. 2002, 2, 1333 10.1021/nl0257519.

[ref114] Zappa D.; Melloni R.; Maraloiu V.-A.; Poli N.; Rizzoni M.; Sberveglieri V.; Sisman O.; Soprani M.; Comini E. Influence of Metal Catalyst on SnO2 Nanowires Growth and Gas Sensing Performance. Proceedings 2017, 1 (4), 460 10.3390/proceedings1040460.

[ref115] Volanti D. P.; Felix A. A.; Orlandi M. O.; Whitfield G.; Yang D.-J.; Longo E.; Tuller H. L.; Varela J. A. The Role of Hierarchical Morphologies in the Superior Gas Sensing Performance of CuO-Based Chemiresistors. Adv. Funct. Mater. 2013, 23 (14), 1759–1766. 10.1002/adfm.201202332.

[ref116] Kim H.; Jin C.; Park S.; Kim S.; Lee C. H2S Gas Sensing Properties of Bare and Pd-Functionalized CuO Nanorods. Sens. Actuators, B 2012, 161 (1), 594–599. 10.1016/j.snb.2011.11.006.

[ref117] Kim Y.-S.; Hwang I.-S.; Kim S.-J.; Lee C.-Y.; Lee J.-H. CuO Nanowire Gas Sensors for Air Quality Control in Automotive Cabin. Sens. Actuators, B 2008, 135 (1), 298–303. 10.1016/j.snb.2008.08.026.

[ref118] Standards - Air Quality - Environment - European Commission http://ec.europa.eu/environment/air/quality/standards.htm (accessed Jan 28, 2020).

[ref119] Ramgir N. S.; Sharma P. K.; Datta N.; Kaur M.; Debnath A. K.; Aswal D. K.; Gupta S. K. Room Temperature H2S Sensor Based on Au Modified ZnO Nanowires. Sens. Actuators, B 2013, 186, 718–726. 10.1016/j.snb.2013.06.070.

[ref120] Lou Z.; Li F.; Deng J.; Wang L.; Zhang T. Branch-like Hierarchical Heterostructure (α-Fe _2_ O _3_ /TiO _2_): A Novel Sensing Material for Trimethylamine Gas Sensor. ACS Appl. Mater. Interfaces 2013, 5 (23), 12310–12316. 10.1021/am402532v. 24102255

[ref121] Xu X.; Sun J.; Zhang H.; Wang Z.; Dong B.; Jiang T.; Wang W.; Li Z.; Wang C. Effects of Al Doping on SnO2 Nanofibers in Hydrogen Sensor. Sens. Actuators, B 2011, 160 (1), 858–863. 10.1016/j.snb.2011.08.072.

[ref122] Krivetsky V.; Ponzoni A.; Comini E.; Rumyantseva M.; Gaskov A. Selective Modified SnO2-Based Materials for Gas Sensors Arrays. Procedia Chem. 2009, 1 (1), 204–207. 10.1016/j.proche.2009.07.051.

[ref123] Arachchige H. M. M. M.; Gunawardhana N.; Zappa D.; Comini E. UV Light Assisted NO2Sensing by SnO2/Graphene Oxide Composite. Proceedings 2018, 2 (13), 787 10.3390/proceedings2130787.

[ref124] Yang D.; Cho I.; Kim D.; Lim M. A.; Li Z.; Ok J. G.; Lee M.; Park I. Gas Sensor by Direct Growth and Functionalization of Metal Oxide/Metal Sulfide Core-Shell Nanowires on Flexible Substrates. ACS Appl. Mater. Interfaces 2019, 11 (27), 24298–24307. 10.1021/acsami.9b06951. 31187618

[ref125] Kolmakov A.; Klenov D. O.; Lilach Y.; Stemmer S.; Moskovits M. Enhanced Gas Sensing by Individual SnO2 Nanowires and Nanobelts Functionalized with Pd Catalyst Particles. Nano Lett. 2005, 5 (4), 667–673. 10.1021/nl050082v. 15826106

[ref126] Wang L.; Wang S.; Xu M.; Hu X.; Zhang H.; Wang Y.; Huang W. A Au-Functionalized ZnO Nanowire Gas Sensor for Detection of Benzene and Toluene. Phys. Chem. Chem. Phys. 2013, 15 (40), 17179–17186. 10.1039/c3cp52392f. 24013527

[ref127] Liu X.; Zhang J.; Yang T.; Guo X.; Wu S.; Wang S. Synthesis of Pt Nanoparticles Functionalized WO3 Nanorods and Their Gas Sensing Properties. Sens. Actuators, B 2011, 156 (2), 918–923. 10.1016/j.snb.2011.03.006.

[ref128] Bhati V. S.; Hojamberdiev M.; Kumar M. Enhanced Sensing Performance of ZnO Nanostructures-Based Gas Sensors: A Review. Energy Reports 2020, 6, 46–62. 10.1016/j.egyr.2019.08.070.

[ref129] Kaur N.; Comini E.; Poli N.; Zappa D.; Sberveglieri G. NiO/ZnO Nanowire-Heterostructures by Vapor Phase Growth for Gas Sensing. Procedia Eng. 2016, 168, 1140–1143. 10.1016/j.proeng.2016.11.376.

[ref130] Raza M. H.; Kaur N.; Comini E.; Pinna N. Toward Optimized Radial Modulation of the Space-Charge Region in One-Dimensional SnO _2_ -NiO Core-Shell Nanowires for Hydrogen Sensing. ACS Appl. Mater. Interfaces 2020, 12 (4), 4594–4606. 10.1021/acsami.9b19442. 31933357

[ref131] Si S.; Li C.; Wang X.; Peng Q.; Li Y. Fe2O3/ZnO Core-Shell Nanorods for Gas Sensors. Sens. Actuators, B 2006, 119 (1), 52–56. 10.1016/j.snb.2005.11.050.

[ref132] Liu Y.; Li G.; Mi R.; Deng C.; Gao P. An Environment-Benign Method for the Synthesis of p-NiO/n-ZnO Heterostructure with Excellent Performance for Gas Sensing and Photocatalysis. Sens. Actuators, B 2014, 191, 537–544. 10.1016/j.snb.2013.10.068.

[ref133] Hwang Y. J.; Boukai A.; Yang P. High Density N-Si/n-TiO _2_ Core/Shell Nanowire Arrays with Enhanced Photoactivity. Nano Lett. 2009, 9 (1), 410–415. 10.1021/nl8032763. 19053790

[ref134] Xu L.; Zheng R.; Liu S.; Song J.; Chen J.; Dong B.; Song H. NiO@ZnO Heterostructured Nanotubes: Coelectrospinning Fabrication, Characterization, and Highly Enhanced Gas Sensing Properties. Inorg. Chem. 2012, 51 (14), 7733–7740. 10.1021/ic300749a. 22747254

[ref135] Wu M.-S.; Chang H.-W. Self-Assembly of NiO-Coated ZnO Nanorod Electrodes with Core-Shell Nanostructures as Anode Materials for Rechargeable Lithium-Ion Batteries. J. Phys. Chem. C 2013, 117 (6), 2590–2599. 10.1021/jp3079327.

[ref136] Yu Q.; Zhu J.; Xu Z.; Huang X. Facile Synthesis of α-Fe2O3@SnO2 Core-Shell Heterostructure Nanotubes for High Performance Gas Sensors. Sens. Actuators, B 2015, 213, 27–34. 10.1016/j.snb.2015.01.130.

[ref137] Choi S.-W.; Katoch A.; Sun G.-J.; Kim J.-H.; Kim S.-H.; Kim S. S. Dual Functional Sensing Mechanism in SnO_2_-ZnO Core-Shell Nanowires. ACS Appl. Mater. Interfaces 2014, 6 (11), 8281–8287. 10.1021/am501107c. 24836937

[ref138] Karnati P.; Akbar S.; Morris P. A. Conduction Mechanisms in One Dimensional Core-Shell Nanostructures for Gas Sensing: A Review. Sens. Actuators, B 2019, 295, 127–143. 10.1016/j.snb.2019.05.049.

