# Supplementary material for: One-Dimensional Nanostructured Oxide Chemoresistive Sensors
Source: Langmuir. 2020 May 26;36(23):6326–44. doi: 10.1021/acs.langmuir.0c00701 (PMC8154880; doi:10.1021/acs.langmuir.0c00701)
Supplement: Supplementary file 1 — la0c00701_si_001.pdf [file la0c00701_si_001.pdf]

Supporting Information

Title: 1-D Nanostructured Oxide Chemoresistive Sensors

*Navpreet Kaur, Mandeep Singh, and Elisabetta Comini**

SENSOR Laboratory, University of Brescia, Via D. Valotti 9, 25133 Brescia, Italy

Conductometric Gas Sensing Measurement Setup

In this section we are briefly describing the gas sensing measurement setup developed and used by the Sensor Lab from last two decades.[1] The conductometric sensing device (figure 3b) were mounted on the TO packages using electro-soldered gold wires. After the device fabrication, the conductometric response of the sensors have been investigated by using a homemade test chamber (Figure S1). Figure 4a shows a real picture of the complete test chamber and figure 4b the inside view of small chamber used to mount and test the sensing device. This test chamber is able to measure up to eight sensors simultaneously. The test equipment consists of a stainless steel chamber (1L volume) placed inside a thermostatic chamber (Binder, Germany model) set at 20 °C to avoid the influence of the external temperature. Humidified air was produced by flowing dry air through a Drechsler bottle, held in a thermostatic bath at 25°C, and then in a

condensation vessel in order to favor the condensation of saturated vapors. The humidified air was mixed with dry air in order to obtain the desired relative humidity (RH) content. The sensor temperatures were controlled by modulating the electric power applied to heaters with Thurlbly-Thandar PL330DP power supplies. A fixed voltage was applied to the sensors (Agilent E3631A power supply), typically 0.1-1 V, measuring the conductance at the same time of each sensor using pico-ammeters (Keithley 486). Gas sensing performances of the metal oxide nanowires were initially screened for the detection of various target gases (reducing and oxidizing gases).

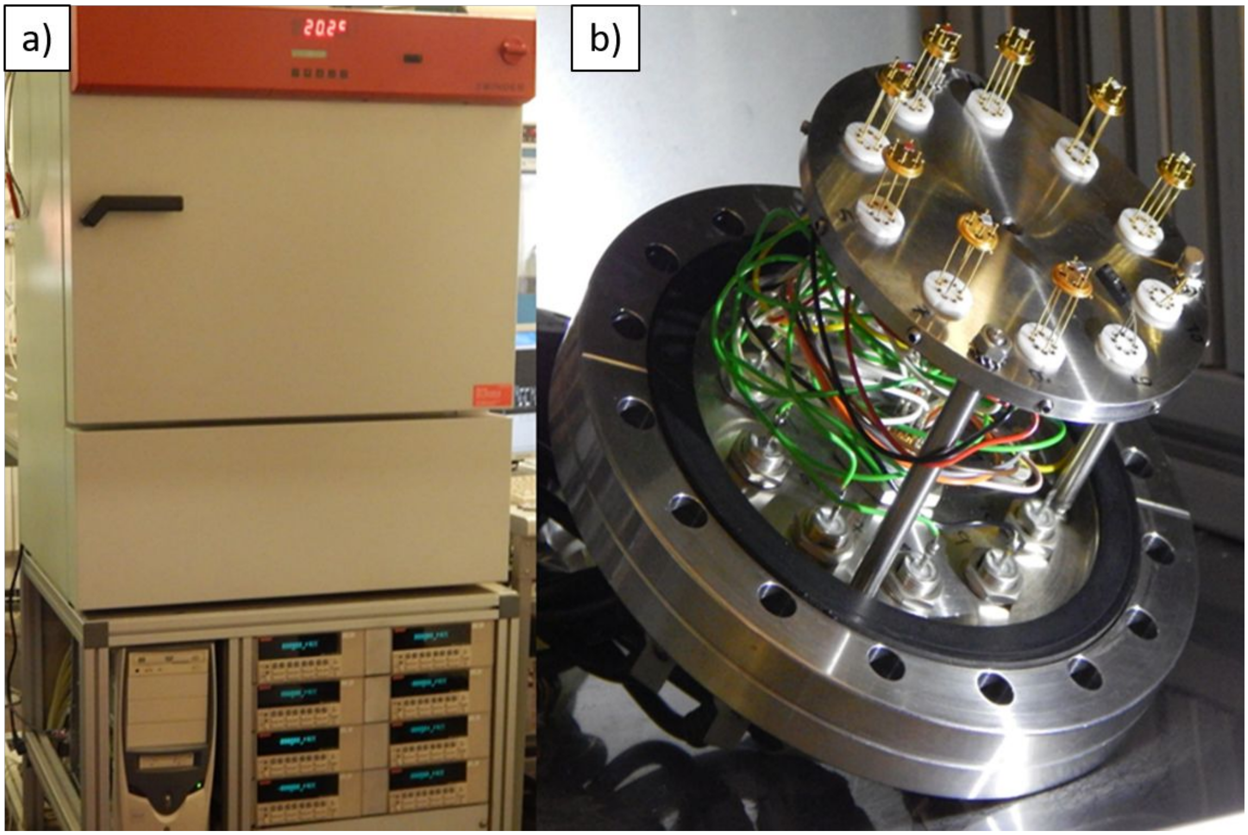

Figure S1. (a) Pictures of the custom measurement chamber used to evaluate the chemical detection performances of fabricated nanostructures at Senor Lab and (b) inside view of small chamber used to mount and test the sensing devices.

Advantages and Disadvantages of Vapor Phase Growth and Thermal Oxidation Growth Techniques

Both VLS/VS mechanism and thermal oxidation for the growth of metal oxides nanowires have been vastly used by Sensor lab and other research groups for gas

sensing applications.[2–5] In order to compare both techniques, we have tabulated (table 1) the key advantages and disadvantages of both growth techniques.

Table S1: Advantages and disadvantages of the VS, VLS and thermal oxidation techniques.

VLS and VS growth		Thermal oxidation	
Advantages	Disadvantages	Advantages	Disadvantages
Growth of highly crystalline nanostructures	Requirement of high evaporation temperature	Low temperature growth	Presence of different defects in the nanostructures
Direct growth on active substrate	Limited selection of substrates due to high temperature	High yield	No large scale production

High yield<br><br>Better reproducibility<br><br>Simple growth setup<br><br>Large scale production	Contamination due to the presence of catalyst alloy in the nanostructures<br><br>Limited control on orientation of nanostructures	Large selection of substrates<br><br>Ease of doping the materials	Presence of metallic residue in the grown nanostructures

Table S2. Comparison of our NWs based chemical/gas sensor performances with literature. Here, ΔG, ΔR and ΔI represent the change in conductance, resistance and

current of a sensor in the presence of gas analyte. While, R_a and R_g =resistance in air and in the presence of gas; I_a and I_g =current in air and in the presence of gas.

SnO_2			
**Technique**	**Morphology**	**Gas/ Concentration**	**Response (S)**
Evaporation-condensation[6]	nanobelts	Ethanol/250 ppm	S=ΔG/G<br>41.6 at 400 °C
VLS mechanism[7]	nanowires	Acetone /100 ppm	S=ΔG/G<br>□30 at 400 °C
Eectrospinning followed by hydrothermal etching[8]	porous nanowires	Ethanol/ 100 ppm	$S=R_a/R_g$<br>□17 at 380 °C

Vapor phase growth method[9]	nanowires	NO_2/5 ppm	$S=R_g/R_a$<br>1909 at 141 °C
Carbothermal reduction process[10]	nanowires	CH_4/400 ppm<br>CO/20 ppm<br>CH_3OH/50 ppm	$S=R_a/R_g$<br>1.20 at 300 °C<br>3.20 at 400 °C<br>6.65 at 400 °C
ZnO			
VLS mechanism[11]	nanowires	Ethanol/500 ppm	$S=\Delta G/G$<br>60 at 400 °C
Thermal Oxidation[12]	nanowires	Ethanol/200 ppm	$R=\Delta G/G$<br>2 at 500 °C
Pulse Laser Deposition[13]	nanorods	Ethanol/500 ppm	$S=R_a/R_g$<br>1.2 at 260 °C

Hydrothermal[14]	nanorods	Ethanol/500 ppm	$S=R_a/R_g$<br>35 at 300 °C
Chemical route[15]	nanopetal	NO_2/20 ppm	$S=R_a/R_g$<br>~119 at RT
vapor phase transport method[16]	nanorods/ nanoflowers	H_2S/ 5 ppm	$S = I_g/I_a$<br>581 at RT
WO_3			
VLS Mechanism[2]	nanowires	H_2S/10 ppm<br>O_3/0.3 ppm	$S=\Delta G/G$<br>104 at 400 °C<br>170 at 200 °C
Thermal Oxidation[17]	nanowires	CO/500 ppm<br>NO_2/9 ppm<br>NH_3/5 ppm	$S=\Delta G/G$<br>68 at 200 °C<br>20 at 300 °C

			10 at 200 °C
Magnetron Sputtering[18]	thin film	NO_2/5 ppm	S=G_a/G_g 8 at 200 °C
Hydrothermal/ impregnation method[19]	nanorods	H_2S/10 ppm	S=R_a/R_g 20 at 250 °C
RF Sputtering[20]	thin film	O_3/0.8 ppm	S=ΔG/G 4.8 at 250 °C
Nanocasting method[21]	mesoporous layer	H_2S/200 ppm	R=R_a/R_g 7 at 125 °C
NiO			
VLS method[22]	nanowires	H_2/500 ppm	S=ΔG/G 80 at 300 °C
Hydrothermal[23]	nanosheets	H_2/500 ppm	S=ΔR/R

			2.25 at 200 °C
VLS method[24]	nanowires	NO_2/1 ppm	S=ΔG/G<br><br>54.8 at 200 °C
RF sputtering[25]	thin film	H_2/500 ppm	S=ΔR/R<br><br>4 at 250 °C
Hydrothermal[26]	nanosheets	NO_2/20 ppm	S= -----not give-----<br><br>0.8 at 250 °C
Chemical reduction[27]	nanowire	NH_3/200 ppm	S=ΔG/G<br><br>0.5 at 400 °C
Hydrothermal[28]	lotus-root slice	ethanol/100 ppm	$S=R_g/R_a$<br><br>6 at 300 °C
CuO			
Thermal oxidation[5]	nanowires	ethanol/500 ppm<br><br>Ozone/300 ppb	S=ΔG/G<br><br>1.7 at 400 °C

			2.5 at 400 °C
Hydrothermal[29]	nanorods	ethanol/1000 ppm	$S=\Delta I/I_g$<br><br>1.7 at 200 °C
Chemical synthesis[30]	nanotubes	CO/100 ppm	$S=R_g/R_a$<br><br>1.55 at 175 °C
Chemical synthesis[30]	nanocubes	CO/100 ppm	$S=R_g/R_a$<br><br>1.13 at 175 °C

Table S3. Comparison of our nano-heterostructures and composite based chemical/gas sensor performances with Literature. Here, ΔG, ΔR and ΔI represent the change in conductance, resistance and current of a sensor in the presence of gas analyte. While, R_a and R_g =resistance in air and in the presence of gas; I_a and I_g =current in air and in the presence of gas.

Sensor Materials	Technique	Morphology	Gas/ Concentration	Response (S)
Nb-WO_3[31]	Thermal oxidation/ Sputtering	nanowires	H_2/ 500 ppm	$S=\Delta G/G$ 10^5 at 200 °C
Pt-NiO[32]	DC reactive magnetron sputtering	Thin film	H_2/ 5000 ppm	$S=I_a/I_g$ 45 at 320 °C
Pt-ZnO[33]	Flame spray pyrolysis	Thin film	C_2H_2/ 1000 ppm	$S=R_a/R_g$ 45 at 300 °C
SnO_2/NiO[34]	Vapor phase growth/ADL	Core/shell nanowires	H_2/ 500 ppm	$S=\Delta G/G$ 114 at 500 °C
NiO/SnO_2 [35]	Electrospinning technique	nanofibers	H_2/500 ppm	$S=R_a/R_g$ 19 at 320 °C
NiO/SnO_2[36]	Hydrothermal	Nano composite	H_2/100 ppm	$S=R_a/R_g$

				45.23 at 450 °C
Fe_2O_3/NiO[37]	Hydrothermal/ pulsed laser deposition	Core/shell nanorods	triethylamine /500 ppm	$S=R_a/R_g$ ☐12 at 40 °C
Fe_2O_3/CuO[37]	Hydrothermal/ pulsed laser deposition	Core/shell nanorods	triethylamine /100 ppm	$S=R_a/R_g$ ☐14 at 40 °C
NiO/ZnO[38]	Vapour phase growth	Branch like heterostructures	Ethanol/ 50 ppm	$S=\Delta G/G$ 6.7 at 400 °C
NiO/ZnO[38]	Vapour phase growth	Branch like heterostructures	Acetone/ 100 ppm	$S=\Delta G/G$ 10 at 400 °C
NiO/ZnO[39]	CBD approach	flower-like structures	Acetone/ 100 ppm	$S=R_a/R_g$ 13 at 330 °C
CuO/NiO[40]	Hydrothermal	Microspheres	H_2S/ 100 ppm	$S=R_g/R_a$ 47.6 at 260 °C

Reference:

(1) Sberveglieri, G.; Faglia, G.; Perego, C.; Nelli, P.; Marks, R. N.; Virgili, T.; Taliani, C.; Zamboni, R. Hydrogen and Humidity Sensing Properties of C60 Thin Films. *Synth. Met.* **1996**, *77* (1–3), 273–275. https://doi.org/10.1016/0379-6779(96)80101-9.

(2) Kaur, N.; Zappa, D.; Poli, N.; Comini, E. Integration of VLS-Grown WO_3 Nanowires into Sensing Devices for the Detection of H_2S and O_3. *ACS Omega* **2019**, *4* (15), 16336–16343. https://doi.org/10.1021/acsomega.9b01792.

(3) Comini, E.; Baratto, C.; Faglia, G.; Ferroni, M.; Vomiero, A.; Sberveglieri, G. Quasi-One Dimensional Metal Oxide Semiconductors: Preparation, Characterization and Application as Chemical Sensors. *Prog. Mater. Sci.* **2009**, *54* (1), 1–67. https://doi.org/10.1016/J.PMATSCI.2008.06.003.

(4) Arafat, M. M.; Haseeb, A. S. M. A.; Dinan, B.; Akbar, S. A. Stress Enhanced TiO2 Nanowire Growth on Ti–6Al–4V Particles by Thermal Oxidation. *Ceram. Int.* **2013**, *39* (6), 6517–6526. https://doi.org/https://doi.org/10.1016/j.ceramint.2013.01.084.

(5) Zappa, D.; Comini, E.; Zamani, R.; Arbiol, J.; Morante, J. R.; Sberveglieri, G. Preparation of Copper Oxide Nanowire-Based Conductometric Chemical Sensors. *Sensors Actuators*

B Chem. **2013**, *182*, 7–15. https://doi.org/10.1016/J.SNB.2013.02.076.

(6) Comini, E.; Faglia, G.; Sberveglieri, G.; Pan, Z.; Wang, Z. L. Stable and Highly Sensitive Gas Sensors Based on Semiconducting Oxide Nanobelts. *Appl. Phys. Lett.* **2002**, *81* (10), 1869–1871. https://doi.org/10.1063/1.1504867.

(7) Zappa, D.; Melloni, R.; Maraloiu, V.-A.; Poli, N.; Rizzoni, M.; Sberveglieri, V.; Sisman, O.; Soprani, M.; Comini, E. Influence of Metal Catalyst on SnO2 Nanowires Growth and Gas Sensing Performance. *Proceedings* **2017**, *1* (4), 460. https://doi.org/10.3390/proceedings1040460.

(8) Li, R.; Chen, S.; Lou, Z.; Li, L.; Huang, T.; Song, Y.; Chen, D.; Shen, G. Fabrication of Porous SnO2 Nanowires Gas Sensors with Enhanced Sensitivity. *Sensors Actuators, B Chem.* **2017**, *252*, 79–85. https://doi.org/10.1016/j.snb.2017.05.161.

(9) Hwang, I. S.; Kim, S. J.; Choi, J. K.; Jung, J. J.; Yoo, D. J.; Dong, K. Y.; Ju, B. K.; Lee, J. H. Large-Scale Fabrication of Highly Sensitive SnO 2 Nanowire Network Gas Sensors by Single Step Vapor Phase Growth. *Sensors Actuators, B Chem.* **2012**, *165* (1), 97–103. https://doi.org/10.1016/j.snb.2012.02.022.

(10) Shehzad, K.; Shah, N. A.; Amin, M.; Abbas, M.; Syed, W. A. Synthesis of SnO_2 Nanowires ForCO, CH_4 and CH_3 OH Gases Sensing. *Int. J. Distrib. Sens. Networks* **2018**, *14* (8), 155014771879075. https://doi.org/10.1177/1550147718790750.

(11) Comini, E.; Faglia, G.; Ferroni, M.; Sberveglieri, G. Gas Sensing Properties of Zinc Oxide Nanostructures Prepared by Thermal Evaporation. *Appl. Phys. A* **2007**, *88* (1), 45–48. https://doi.org/10.1007/s00339-007-3978-9.

(12) Zappa, D.; Comini, E.; Sberveglieri, G. Thermally Oxidized Zinc Oxide Nanowires for Use as Chemical Sensors. *Nanotechnology* **2013**, *24* (44), 444008. https://doi.org/10.1088/0957-4484/24/44/444008.

(13) Cao, P.; Yang, Z.; Navale, S. T.; Han, S.; Liu, X.; Liu, W.; Lu, Y.; Stadler, F. J.; Zhu, D. Ethanol Sensing Behavior of Pd-Nanoparticles Decorated ZnO-Nanorod Based Chemiresistive Gas Sensors. *Sensors Actuators B Chem.* **2019**, *298*, 126850. https://doi.org/10.1016/J.SNB.2019.126850.

(14) Zhu, L.; Li, Y.; Zeng, W. Enhanced Ethanol Sensing and Mechanism of Cr-Doped ZnO Nanorods: Experimental and Computational Study. *Ceram. Int.* **2017**, *43* (17), 14873–14879. https://doi.org/10.1016/J.CERAMINT.2017.08.003.

(15) Sonker, R. K.; Sabhajeet, S. R.; Singh, S.; Yadav, B. C. Synthesis of ZnO Nanopetals and Its Application as NO2 Gas Sensor. *Mater. Lett.* **2015**, *152*, 189–191. https://doi.org/10.1016/j.matlet.2015.03.112.

(16) Hosseini, Z. S.; Zad, A. I.; Mortezaali, A. Room Temperature H2S Gas Sensor Based on Rather Aligned ZnO Nanorods with Flower-like Structures. *Sensors Actuators, B Chem.* **2015**, *207* (PartA), 865–871. https://doi.org/10.1016/j.snb.2014.10.085.

(17) Zappa, D.; Bertuna, A.; Comini, E.; Molinari, M.; Poli, N.; Sberveglieri, G. Tungsten Oxide Nanowires for Chemical Detection. *Anal. Methods* **2015**, *7* (5), 2203–2209. https://doi.org/10.1039/C4AY02637C.

(18) Parellada-Monreal, L.; Gherardi, S.; Zonta, G.; Malagù, C.; Casotti, D.; Cruciani, G.; Guidi, V.; Martínez-Calderón, M.; Castro-Hurtado, I.; Gamarra, D.; Lozano, J.; Presmanes, L.; Mandayo, G. G. WO3 Processed by Direct Laser Interference Patterning for NO2 Detection. *Sensors Actuators B Chem.* **2020**, *305*, 127226. https://doi.org/10.1016/J.SNB.2019.127226.

(19) Kruefu, V.; Wisitsoraat, A.; Tuantranont, A.; Phanichphant, S. Ultra-Sensitive H2S Sensors Based on Hydrothermal/Impregnation-Made Ru-Functionalized WO3 Nanorods. *Sensors Actuators B Chem.* **2015**, *215*, 630–636. https://doi.org/10.1016/J.SNB.2015.03.037.

(20) Belkacem, W.; Labidi, A.; Guérin, J.; Mliki, N.; Aguir, K. Cobalt Nanograins Effect on the Ozone Detection by WO3 Sensors. *Sensors Actuators B Chem.* **2008**, *132* (1), 196–201. https://doi.org/10.1016/J.SNB.2008.01.023.

(21) Wang, Y.; Liu, J.; Cui, X.; Gao, Y.; Ma, J.; Sun, Y.; Sun, P.; Liu, F.; Liang, X.; Zhang, T.; Lu, G. NH3 Gas Sensing Performance Enhanced by Pt-Loaded on Mesoporous WO3. *Sensors Actuators, B Chem.* **2017**, *238*, 473–481. https://doi.org/10.1016/j.snb.2016.07.085.

(22) Kaur, N.; Comini, E.; Zappa, D.; Poli, N.; Sberveglieri, G. Nickel Oxide Nanowires: Vapor Liquid Solid Synthesis and Integration into a Gas Sensing Device. *Nanotechnology* **2016**, *27* (20), 205701. https://doi.org/10.1088/0957-4484/27/20/205701.

(23) Tong, P. Van; Hoa, N. D.; Duy, N. Van; Quang, V. Van; Lam, N. T.; Hieu, N. Van. In-Situ Decoration of Pd Nanocrystals on Crystalline Mesoporous NiO Nanosheets for Effective Hydrogen Gas Sensors. *Int. J. Hydrogen Energy* **2013**, *38* (27), 12090–12100. https://doi.org/10.1016/J.IJHYDENE.2013.06.120.

(24) Kaur, N.; Zappa, D.; Comini, E. Shelf Life Study of NiO Nanowire Sensors for NO2 Detection. *Electron. Mater. Lett.* **2019**, *15* (6), 743–749. https://doi.org/10.1007/s13391-019-00172-5.

(25) Chou, P.-C.; Chen, H.-I.; Liu, I.-P.; Chen, C.-C.; Liou, J.-K.; Hsu, K.-S.; Liu, W.-C. Hydrogen Sensing Performance of a Nickel Oxide (NiO) Thin Film-Based Device. *Int. J. Hydrogen Energy* **2015**, *40* (1), 729–734. https://doi.org/10.1016/J.IJHYDENE.2014.10.142.

(26) Hoa, N. D.; El-Safty, S. A. Synthesis of Mesoporous NiO Nanosheets for the Detection of Toxic NO2 Gas. *Chem. - A Eur. J.* **2011**, *17* (46), 12896–12901. https://doi.org/10.1002/chem.201101122.

(27) Wang, J.; Wei, L.; Zhang, L.; Jiang, C.; Siu-Wai Kong, E.; Zhang, Y. Preparation of High Aspect Ratio Nickel Oxide Nanowires and Their Gas Sensing Devices with Fast Response and High Sensitivity. *J. Mater. Chem.* **2012**, *22* (17), 8327. https://doi.org/10.1039/c2jm16934g.

(28) Cao, S.; Peng, L.; Liu, B.; Han, T.; Zhao, C.; Zhu, D.; Tang, Y. Hydrothermal Synthesis of Novel Lotus-Root Slice NiO Architectures with Enhanced Gas Response Properties. *J. Alloys Compd.* **2019**, *798*, 478–483. https://doi.org/10.1016/J.JALLCOM.2019.05.267.

(29) Sarıca, N.; Alev, O.; Arslan, L. Ç.; Öztürk, Z. Z. Characterization and Gas Sensing Performances of Noble Metals Decorated CuO Nanorods. *Thin Solid Films* **2019**, *685*, 321–328. https://doi.org/10.1016/J.TSF.2019.06.046.

(30) Hou, L.; Zhang, C.; Li, L.; Du, C.; Li, X.; Kang, X.-F.; Chen, W. CO Gas Sensors Based on P-Type CuO Nanotubes and CuO Nanocubes: Morphology and Surface Structure Effects on the Sensing Performance. *Talanta* **2018**, *188*, 41–49. https://doi.org/10.1016/j.talanta.2018.05.059.

(31) Zappa, D. The Influence of Nb on the Synthesis of WO3 Nanowires and the Effects on Hydrogen Sensing Performance. *Sensors* **2019**, *19* (10), 2332. https://doi.org/10.3390/s19102332.

(32) Hotovy, I.; Huran, J.; Siciliano, P.; Capone, S.; Spiess, L.; Rehacek, V. Enhancement of H2 Sensing Properties of NiO-Based Thin Films with a Pt Surface Modification. In *Sensors and Actuators, B: Chemical*; Elsevier, 2004; Vol. 103, pp 300–311. https://doi.org/10.1016/j.snb.2004.04.109.

(33) Tamaekong, N.; Liewhiran, C.; Wisitsoraat, A.; Phanichphant, S. Acetylene Sensor Based on Pt/ZnO Thick Films as Prepared by Flame Spray Pyrolysis. *Sensors Actuators, B Chem.* **2011**, *152* (2), 155–161. https://doi.org/10.1016/j.snb.2010.11.058.

(34) Raza, M. H.; Kaur, N.; Comini, E.; Pinna, N. Toward Optimized Radial Modulation of the Space-Charge Region in One-Dimensional SnO_2–NiO Core–Shell Nanowires for Hydrogen Sensing. *ACS Appl. Mater. Interfaces* **2020**, *12* (4), 4594–4606. https://doi.org/10.1021/acsami.9b19442.

(35) Wang, Z.; Li, Z.; Sun, J.; Zhang, H.; Wang, W.; Zheng, W.; Wang, C. Improved Hydrogen Monitoring Properties Based on P-NiO/n-SnO2 Heterojunction Composite Nanofibers. *J. Phys. Chem. C* **2010**, *114* (13), 6100–6105. https://doi.org/10.1021/jp9100202.

(36) Wei, C.; Bo, B.; Tao, F.; Lu, Y.; Peng, S.; Song, W.; Zhou, Q. Hydrothermal Synthesis and Structural Characterization of NiO/SnO_2 Composites and Hydrogen Sensing Properties. *J. Spectrosc.* **2015**, *2015*, 1–6. https://doi.org/10.1155/2015/450485.

(37) Xu, Q.; Zhang, Z.; Song, X.; Yuan, S.; Qiu, Z.; Xu, H.; Cao, B. Improving the Triethylamine Sensing Performance Based on Debye Length: A Case Study on A-Fe2O3@NiO(CuO) Core-Shell Nanorods Sensor Working at near Room-Temperature. *Sensors Actuators, B Chem.* **2017**, *245*, 375–385. https://doi.org/10.1016/j.snb.2017.01.136.

(38) Kaur, N.; Zappa, D.; Ferroni, M.; Poli, N.; Campanini, M.; Negrea, R.; Comini, E. Branch-like NiO/ZnO Heterostructures for VOC Sensing. *Sensors Actuators B Chem.* **2018**, *262*, 477–485. https://doi.org/10.1016/J.SNB.2018.02.042.

(39) Liu, Y.; Li, G.; Mi, R.; Deng, C.; Gao, P. An Environment-Benign Method for the Synthesis of p-NiO/n-ZnO Heterostructure with Excellent Performance for Gas Sensing and Photocatalysis. *Sensors Actuators B Chem.* **2014**, *191*, 537–544. https://doi.org/10.1016/J.SNB.2013.10.068.

(40) Wang, Y.; Qu, F.; Liu, J.; Wang, Y.; Zhou, J.; Ruan, S. Enhanced H2S Sensing Characteristics of CuO-NiO Core-Shell Microspheres Sensors. *Sensors Actuators, B Chem.* **2015**, *209*, 515–523. https://doi.org/10.1016/j.snb.2014.12.010.
